# Nucleotide diversity is a poor predictor of short-term adaptive potential

**DOI:** 10.1073/pnas.2536181123

**Published:** 2026-06-29

**Authors:** Katie L. Abson, Lillith Zijmers, Elizabeth A. Mittell, Euan A. Young, Erik Postma, Adam Eyre-Walker, Jarrod D. Hadfield

**Affiliations:** ^a^ Institute of Ecology and Evolution, School of Biological Sciences, University of Edinburgh, Edinburgh EH9 3FL, United Kingdom; ^b^ School of Life Sciences, University of Sussex, Brighton BN1 9QG, United Kingdom; ^c^ Groningen Institute for Evolutionary Life Sciences, University of Groningen, Groningen 9747 AG, The Netherlands; ^d^ Centre for Ecology and Conservation, University of Exeter, Penryn TR10 9FE, United Kingdom

**Keywords:** adaptive potential, nucleotide diversity, genetic variation, evolvability, conservation genetics

## Abstract

The capacity to adapt is essential for a population to avoid extinction in a changing world and is recognized as a global conservation priority. Adaptation requires additive (heritable) genetic variation for traits that influence survival and fecundity, but measuring this variation is difficult, particularly in species of conservation concern. Instead, molecular genetic diversity is often used to infer adaptive potential. However, previous research has cast doubt on the suitability of traditional molecular markers (allozymes and microsatellites) for this purpose given their weak relationship with heritability—a common measure of additive genetic variance. Advances in sequencing technology have since shifted focus toward nucleotide diversity and variation in functional regions, but their practicality for predicting adaptive potential remains debated. Furthermore, heritability itself is a poor proxy for adaptive potential because it depends on environmental variance. We collated 2,113 published estimates of evolvability—a measure of additive genetic variance that avoids environmental confounding—across 193 eukaryotic species, and evaluated how well evolvability is predicted by molecular diversity. We find that microsatellite and nucleotide diversity are not significantly correlated to each other, and neither predict evolvability. Nucleotide diversity explains 1.1% of interspecific differences in evolvability and doubling nucleotide diversity only corresponds to a 11.7% increase in evolvability. With theoretical work, we show that such weak associations are expected. Together, our results suggest that simple molecular measures of genetic variation are insufficient for predicting adaptive potential and reliance on these metrics risks misinforming conservation management.

## Significance

The current paradigm in conservation genetics suggests that species with the lowest molecular genetic diversity have the lowest capacity to adapt. Despite previous concerns that traditional measures of molecular genetic variation are not useful predictors of adaptive potential, the conflation of genetic diversity and adaptive potential remains prevalent in both scientific literature and global policy. By combining theory with a large dataset of genetic variation across hundreds of species, we show that molecular sequence variation is weakly related to the level of heritable variation in traits across species. This demonstrates that genetic diversity does not reliably predict adaptive potential in the short-term, and highlights the urgent need to move beyond simple measures when assessing the evolutionary resilience of populations.

Genetic diversity has played an important role in conservation genetics since the emergence of the field more than 50 y ago, where it is often used as an indicator of a population’s genetic health (1, 2). Beyond the challenges of demographic and environmental stochasticity, small populations face an elevated risk of extinction due to the genetic consequences of their reduced size (3). Following a bottleneck, the mean fitness of a population may be reduced by inbreeding depression, where the effects of recessive deleterious variants are expressed more frequently due to increases in homozygosity. Over subsequent generations, fitness can further decline as deleterious variants stochastically increase to fixation. Finally, the genetic health of a population can be compromised by a reduction in its capacity to adapt to a change in the environment. Initially, adaptation is expected to occur mainly through changes in allele frequencies of standing genetic variation, since beneficial mutations are rare and their initial increase in frequency is slow and subject to high rates of stochastic loss (4). Therefore, it is generally believed that loss of genetic diversity limits adaptive potential and, consequently, that small populations are less robust to environmental change. Reflecting this view, 196 countries have committed to maintaining genetic diversity within and between populations to safeguard adaptive potential, a critical conservation target of the Kunming-Montreal Global Biodiversity Framework (5). However, there is no consensus on which aspects of genetic variation are essential to preserve.

Classical theory assumes that most genetic variants that will become adaptive following a change in environment currently have negligible fitness effects (6–9). Therefore, adaptive potential has traditionally been inferred from the genetic diversity of presumed neutral molecular markers, such as microsatellites or nucleotide polymorphisms in noncoding regions (2). Conversely, some argue that this approach is unhelpful and that functional variation should be prioritized instead (10). In practice, identifying functionally important loci is difficult, and prioritizing the diversity of a limited subset of functional loci may be counterproductive in the long term as we cannot be certain which genetic variants will be beneficial in future environments (11). Therefore, others maintain that preserving genome-wide diversity, including neutral sites, remains crucial (11–13).

Adaptive potential is often defined loosely as “the capacity to adapt,” which does not provide a useful framework for assessment and conservation. A precise and practical definition of adaptive potential is the rate at which fitness can increase in one generation due to selection (14, 15), which is equal to the amount of additive (heritable) genetic variance for relative fitness, $V_{A}\left(w\right)$ (Fisher’s Fundamental Theorem of Natural Selection: 16). While $V_{A}\left(w\right)$ may not necessarily translate into observed increases in mean fitness because other factors such as density dependence can obscure or counteract genetic gains (17), it is a valuable standardized metric: populations with higher genetic variance for fitness have the capacity to evolve more rapidly in response to selection. Of course, it is impossible to know the amount of $V_{A}\left(w\right)$ that a population will have at the time of a future environmental change, and even accurately estimating current levels of $V_{A}\left(w\right)$ is difficult as it requires fitness measures for a large number of known relatives (18). For this reason, direct estimates of $V_{A}\left(w\right)$ exist for only a limited number of species (19, 20) and rarely for those of conservation concern (but see ref. 21). Ultimately, $V_{A}\left(w\right)$ is determined by the strength of selection operating on the traits that influence survival and fecundity and their genetic (co)variances (22, 23). Therefore, if we assume that future environmental changes induce directional selection in trait space that is random and uniformly distributed, then the average additive genetic variance ($V_{A}$) across traits is equal to $V_{A}\left(w\right)$ (24). This means that $V_{A}\left(w\right)$, and therefore adaptive potential, can be approximated by the mean $V_{A}$ across a random sample of traits (14), though this would still require more data than is typically feasible for species of conservation concern.

Molecular genetic diversity is an appealing proxy for adaptive potential because it is relatively easy and inexpensive to measure (2) and, under some quantitative genetic theory, genome-wide genetic diversity is expected to scale proportionally with $V_{A}\left(w\right)$ (25). However, this expectation rests on several stringent assumptions—most critically that allele frequency is independent of its effect (25). Under models of mutation–selection balance, allele frequencies and effects are negatively related as alleles with larger deleterious effects are kept at lower frequencies (26), consistent with patterns observed in the genetic architecture of human complex traits (27). Therefore, in reality, the relationship between genome-wide genetic diversity and $V_{A}\left(w\right)$ could be much weaker, particularly if alleles that are currently deleterious contribute to a future adaptive response (10).

At present, the true relationship between $V_{A}\left(w\right)$ and molecular genetic diversity cannot be directly assessed because there are so few estimates of both measures within the same population or species. Instead, this relationship can be inferred by testing how strongly genetic diversity predicts $V_{A}$ of traits. Using this approach, two previous studies have found that the relationship between traditional molecular measures (allozyme and microsatellite diversity) and mean $V_{A}$ across traits is weak (14, 28). However, it is unclear whether these findings are informative to contemporary conservation genetic practices, where measuring diversity of nucleotide sites is increasingly favored (2). First, nucleotide diversity is weakly correlated with allozyme diversity across species (29). To our knowledge, no broad cross-species comparisons have been reported between nucleotide and microsatellite diversity, and the associations reported within species are inconsistent and typically based on very few populations (30–32); below, we show that there is no detectable relationship between microsatellite and nucleotide diversity across a larger sample of species (Fig. 3). Second, both of these earlier studies (14, 28) assessed how well genetic diversity predicts variance-standardized $V_{A}$, “heritability” or $h^{2}$. Although it is the most widely reported standardization of $V_{A}$, $h^{2}$ is a poor comparative measure due to its dependence on environmental and nonadditive genetic variance (33, 34), which may contribute to the weak correlation between heritability and molecular diversity (28) or population size (35). Instead, the mean-standardized $V_{A}$, “evolvability” or $I_{A}$, has been proposed as a more suitable quantitative genetic measure of adaptive potential (33, 34). $I_{A}$ quantifies the maximum proportional change in the trait mean per generation under a standardized strength of selection (equivalent to selection acting on fitness itself), and unlike $h^{2}$, $I_{A}$ is unaffected by nonadditive genetic and environmental variance. Like the different measures of molecular diversity, $I_{A}$ and $h^{2}$ are only weakly correlated with each other, particularly in natural populations (33, 34, 36, 37). Crucially, this means that the weak relationships that have previously been reported between allozyme or microsatellite diversity and heritability do not necessarily imply that nucleotide diversity is a poor measure of adaptive potential.

Limited data availability previously prevented the assessment of whether nucleotide diversity predicts a species’ mean evolvability of traits (14) and the nature of the relationship between molecular genetic variation and evolvability remains a key question in evolutionary biology (38). In light of the global recognition of the importance of adaptive potential, and ongoing debate regarding the utility of different genetic measures for predicting it, addressing this question is now essential. Here, we compile thousands of published estimates of quantitative (evolvability, $I_{A}$ and heritability, $h^{2}$) and molecular (pairwise nucleotide diversity, π and microsatellite expected heterozygosity, $H_{e}$) genetic variation across 246 eukaryotic species (Table 1). We evaluate how reliably molecular genetic measures predict adaptive potential, first by quantifying the observed relationships between molecular and quantitative genetic variation across species, and then using theoretical models to assess the expected strength of these associations under various evolutionary conditions.

**Table 1.** Summary of sample sizes for main analyses of mean-scaled additive genetic variance (evolvability) and variance-scaled additive genetic variance (heritability)

Molecular marker	Diversity level	Evolvability ($I_{A}$)			Heritability ($h^{2}$)		
		Estimates	Publications	Species	Estimates	Publications	Species
Nucleotide (π)	Same species	1,373	197	108	2,434	293	130
	Same population	69	7	6	101	9	7
Nucleotide ($\pi_{N}/\pi_{S}$)	Same species	796	100	44	1,448	151	49
	Same population	2	1	1	14	2	2
Microsatellite ($H_{e}$)	Same species	860	150	65	1,692	241	75
	Same population	508	91	40	1,090	152	46
All data		2,113	309	193	3,693	473	246

Sample sizes for the univariate analyses were dependent on the availability of estimates of molecular diversity (π= pairwise nucleotide diversity, $\pi_{N}/\pi_{S}$= ratio of nonsynonymous to synonymous pairwise nucleotide diversity, $H_{e}$= expected heterozygosity) from the same species; the number of evolvability/heritability estimates where a corresponding molecular measure was available from the same population is indicated. Bivariate analyses used all available quantitative genetic data.

## Results

### Nucleotide Diversity vs. Evolvability.

**Fig. 1.**
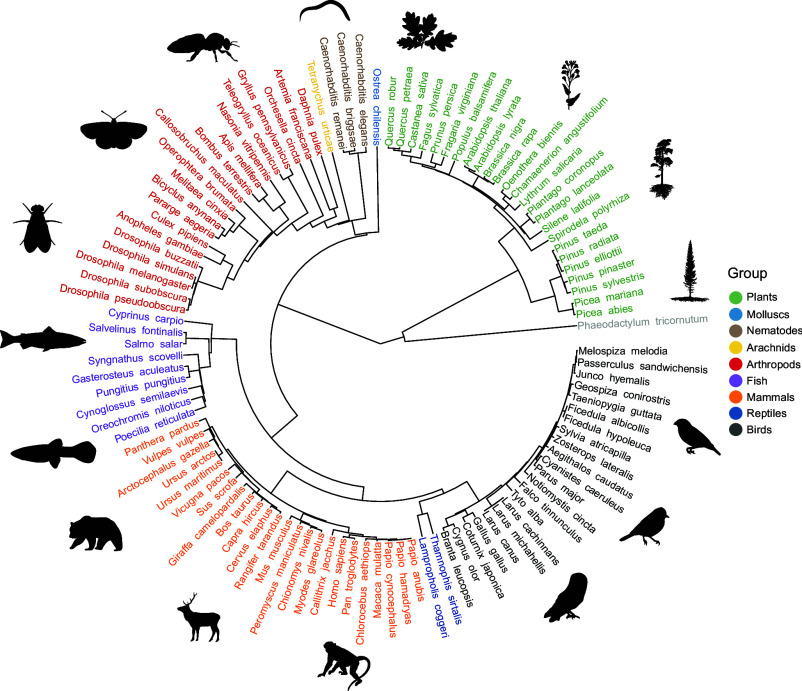
Phylogeny used in the univariate analysis of evolvability with nucleotide diversity as a predictor, visualized with ggtree v3.10.1 (39) and rphylopic v1.5.0 (40).

**Fig. 2.**
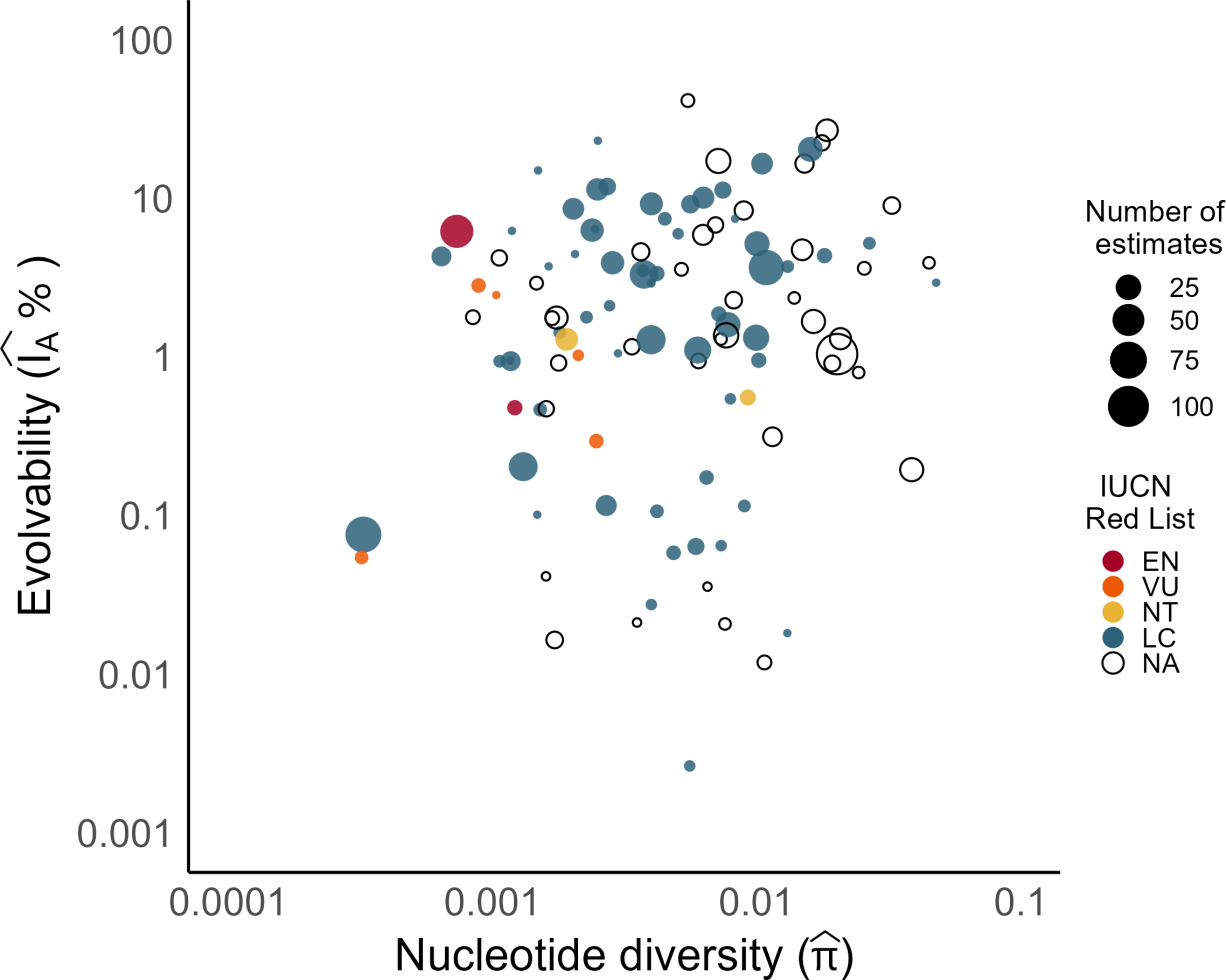
Average *measured* mean-scaled additive genetic variance (evolvability, $\hat{I_{A}}$) vs. neutral pairwise nucleotide diversity ($\hat{\pi }$) assayed in the same species (n=108), excluding nonpositive estimates. Both measures are plotted on log_10_ scale, though data were log_*e*_-transformed for analyses. The size of the points shows the number of estimates over which the average evolvability is calculated and the color shows the IUCN Red List (41) category for that species, where EN = Endangered, VU = Vulnerable, NT = Near threatened, LC = Least concern, and NA = Not classified.

To investigate the relationship between measured genome-wide nucleotide diversity, $\hat{\pi }$, and *true* average trait evolvability, $I_{A}$, we compiled 1,373 published estimates of evolvability from 108 species for which measures of putatively neutral nucleotide diversity were also available (Fig. 1). Using univariate linear mixed models that control for phylogenetic relatedness and differences in estimation between evolvability estimates, we found no meaningful association between $ln(I_{A})$ and $ln(\hat{\pi })$ (Fig. 2). Estimated parameters are summarized by their posterior median and 95% credible intervals. The slope of the regression was β=0.160[−0.125−0.452](P=0.282) and despite substantial variation in $ln(I_{A})$ between-species (SD of 1.563 [0.811 − 2.510]), only $R^{2}=1.1\%\left[-2.1-8.7\right]$ of this could be predicted by $ln(\hat{\pi })$ (note we carry the sign in the $R^{2}$ value). We also calculated $2^{\beta }$, a measure of the relationship that is independent of the amount of interspecific variation in $ln(I_{A})$ (*Materials and Methods*), which suggests that doubling $\hat{\pi }$ would only increase mean evolvability by 11.7% [−9.1 − 35.9]. There was substantial variation in $ln(\hat{I_{A}})$ within-species, some of which can be explained by methodological or trait differences (*SI Appendix*, Tables S5 and S6). An equivalent bivariate model, which leverages information from 85 additional species with available estimates of $I_{A}$ but not $\hat{\pi }$, estimated a similarly weak association between $ln(I_{A})$ and $ln(\hat{\pi })$ (β=−0.047[−0.629−0.586],P=0.877, $2^{\beta }=-3.2\%\left[-41.1-41.6\right]$ and $R^{2}=-0.2\%\left[-20.6-14.6\right]$). $ln(\hat{\pi })$ was also weakly predictive of species’ average heritability (*SI Appendix*, Fig. S3).

Some authors suggest that functional variation is more critical for conservation of adaptive potential (10). Nonsynonymous diversity, $\pi_{N}$, is a relatively easy measure of putatively functional (selected) variation but is strongly correlated with neutral π (42). We therefore repeated the univariate and bivariate analyses, substituting genome-wide diversity $ln(\hat{\pi })$ for the ratio of nonsynonymous to synonymous diversity, $ln({\hat{\pi }}_{N}/{\hat{\pi }}_{S})$, as an alternative simple metric which depends on both effective population size, $N_{e}$, and the average strength of selection (43). The credible intervals were wider than those of the equivalent analysis of neutral nucleotide diversity, but point estimates were similarly weak. The slope of the regression was β=0.014[−0.715−0.685](P=0.974), therefore doubling ${\hat{\pi }}_{N}/{\hat{\pi }}_{S}$ only corresponds to an increase in evolvability of 1.0% [−39.1 − 60.8], and 0.0% [−9.9 − 20.2] of variation in $ln(I_{A})$ could be predicted by $ln({\hat{\pi }}_{N}/{\hat{\pi }}_{S})$. The equivalent bivariate model estimated a stronger association between $ln(I_{A})$ and $ln({\hat{\pi }}_{N}/{\hat{\pi }}_{S})$ but with wide credible intervals (β=0.642[−0.162−1.502],P=0.140, $2^{\beta }=56.1\%\left[-22.7-154.9\right]$ and $R^{2}=14.3\%\left[-2.2-37.0\right]$). $ln({\hat{\pi }}_{N}/{\hat{\pi }}_{S})$ was also weakly predictive of species-mean $h^{2}$ (*SI Appendix*, Fig. S3).

### Microsatellite Diversity vs. Evolvability.

**Fig. 3.**
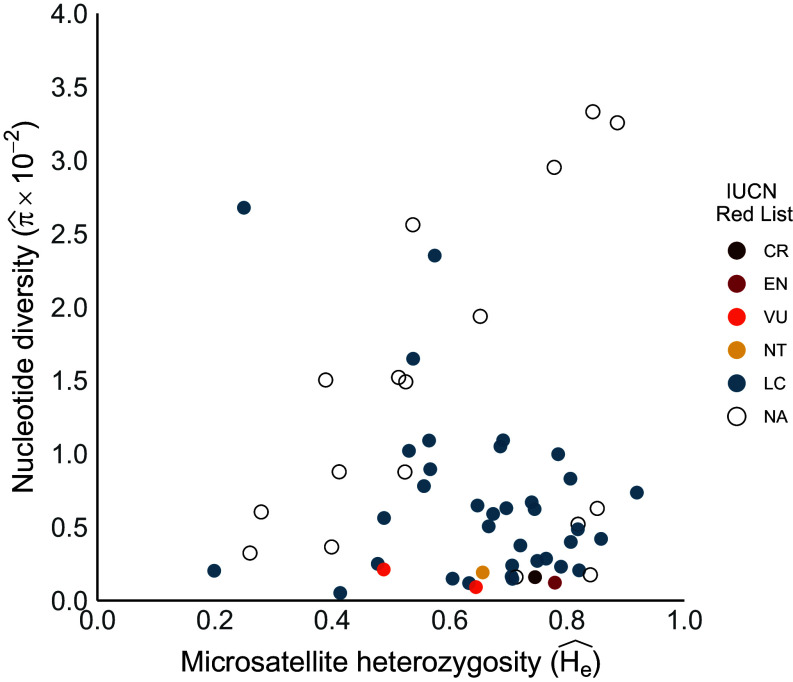
Mean neutral pairwise nucleotide diversity ($\hat{\pi }$) vs. microsatellite expected heterozygosity ($\hat{H_{e}}$) across 57 eukaryotic species. Color shows the IUCN Red List category for the species, where CR = Critically endangered, EN = Endangered, VU = Vulnerable, NT = Near threatened, LC = Least concern, and NA = Not classified.

Although used less frequently, microsatellites are still popular markers of choice in conservation genetics (2). We estimated the correlation between mean nucleotide $\hat{\pi }$ and mean microsatellite expected heterozygosity, $\hat{H_{e}}$, across 57 species and found it to be weak and nonsignificant (r=−0.021[−0.280−0.238],P=0.867; Fig. 3). Therefore, microsatellites and nucleotides may differ in their ability to predict adaptive potential. Previous work has shown that microsatellite diversity weakly predicts $h^{2}$ (14). However, if $I_{A}$ is a better proxy for adaptive potential and $h^{2}$ and $I_{A}$ are only weakly correlated, microsatellite diversity may be a better predictor of adaptive potential than this earlier study suggests. A weak relationship between $h^{2}$ and $I_{A}$ has been found across traits (33, 34, 36)—a result we also observe here with a bivariate model of 1,976 $h^{2}$ and $I_{A}$ estimates ($r=0.135\left[0.077-0.196\right],P<0.5\times 10^{-3}$; *SI Appendix*, Fig. S2). We additionally show that across 183 species, average levels of $\hat{h^{2}}$ and $\hat{I_{A}}$ are also weakly and not significantly correlated (r=0.130[−0.085−0.366],P=0.271). Nevertheless, when we used the univariate model described above with $ln(\hat{\pi })$ substituted for $ln(\hat{H_{e}})$, the regression of $ln(I_{A})$ on $ln(\hat{H_{e}})$ was also found to be weak (β=−0.473[−1.334−0.258],P=0.239), therefore doubling $\hat{H_{e}}$ may reduce mean evolvability by −28.0%[−62.0−16.7]). The $R^{2}$ was effectively zero (−0.8%[−10.4−1.1]).

Since our dataset contains more data for species with both $h^{2}$ and microsatellite diversity than was previously available (14)—specifically, 49% of $h^{2}$ estimates were not included in the previous study and a further 13 species are represented—we also quantified the regression of $h^{2}$ on $ln(\hat{H_{e}})$. Consistent with the findings of 14, initial models found that $ln(\hat{H_{e}})$ was predictive of $h^{2}$ ($\beta =0.088\left[0.066-0.110\right],P<0.5\times 10^{-3}$ and $R^{2}=4.3\%\left[0.4-9.1\right]$), but this association was largely driven by a single study on *Arabidopsis thaliana* (44). When data from this study were excluded, the estimated relationship was weaker (β=−0.030[−0.124−0.061],P=0.534 and $R^{2}=-0.5\%\left[-9.5-4.9\right]$). Details of these additional analyses are provided in *SI Appendix* and sample sizes are shown in Table 1.

#### Precision of estimates.

Our average nucleotide diversity estimates ($\hat{\pi }$) come with error and are generally from different populations than those assayed for quantitative genetic variation (Table 1). By analyzing individual population-level estimates ($\tilde{\pi }$) that contribute to average $\hat{\pi }$, we estimate the true between-species and between-population variance in π to be 1.166 [0.913 − 1.433]$\times 10^{-4}$ and 0.072 [0.024 − 0.119]$\times 10^{-4}$, respectively. The estimation error variance on $\tilde{\pi }$ was estimated to be 0.105 [0.064 − 0.152]$\times 10^{-4}$. From these variances, and accounting for differently weighted contributions of $\tilde{\pi }$ to $\hat{\pi }$, we estimate that $\hat{\pi }$ strongly predicts π in a new population ($R_{\pi_{-},\hat{\pi }}^{2}=0.852\left[0.801-0.900\right]$) and the mean π of a species ($R_{\bar{\pi },\hat{\pi }}^{2}=0.903\left[0.876-0.925\right]$). The $R^{2}$ and β reported for the main analyses are expected to be attenuated by these two factors, respectively. Adjusting for this attenuation gives an $R^{2}$ of 1.3% compared to 1.1% and a $2^{\beta }$ of 13.1% compared to 11.7%. The conditions under which error in $\hat{I_{A}}$ biases estimates of β or $R^{2}$ are much more restrictive, and we therefore expect there to be little to no impact (see *SI Appendix* for details).

### Theoretical Expectations.

To determine whether the observed empirical relationships are consistent with theoretical expectations, we derived the expected regression slope of $ln(V_{A})$ on ln(π) under several models of mutation–selection-drift balance. For neutral traits at equilibrium a one-to-one relationship between $ln(V_{A})$ and ln(π) is expected with β=1, unless π strongly covaries with features of the genetic architecture underlying the trait (the number and length of independent loci and the effect of a new mutation). It has been suggested that departures from equilibrium (i.e., following a population bottleneck or expansion) may decouple the relationship between $V_{A}$ and π (14, 45–47) because mutation replenishes quantitative genetic variation far faster ($10^{-3}$ to $10^{-2}$ per trait per generation, 48) than nucleotide diversity ($10^{-9}$ to $10^{-8}$ per site per generation, 49). However, we show that even under nonequilibrium conditions a one-to-one relationship is expected under neutrality if $V_{A}$ and π have been at equilibrium at some point previously (*SI Appendix*, Fig. S7). External perturbations to allele frequencies (e.g. a translocation event) are required to cause the relationship to deviate from one-to-one.

**Fig. 4.**
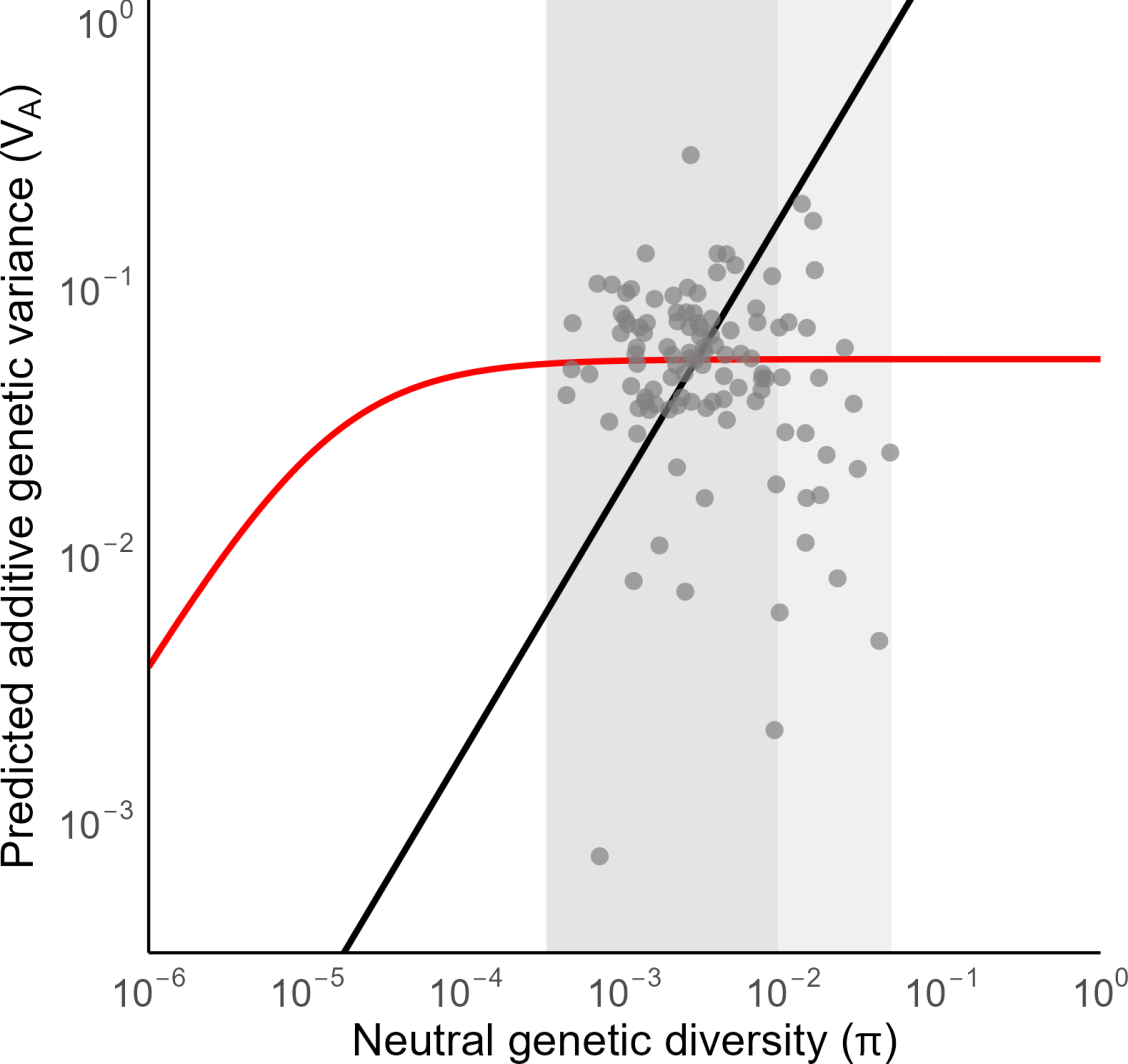
The predicted relationship between additive genetic variance ($V_{A}$) and neutral genetic diversity ($\pi =4N_{e}\mu$) at equilibrium under the finite-population House-of-Cards approximation (50) with an average selection coefficient of s=−0.001. Circles show the predicted $V_{A}$ for 110 multicellular eukaryotic species calculated from empirical estimates of their effective population size, $N_{e}$, and per-site mutation rate, μ, reported by ref. 51. Lines show the expected relationship under two extreme scenarios in which variation in π is driven i) only by changes in μ, with $N_{e}$ fixed at the median empirical value of $10^{5}$ (black), or ii) only by changes in $N_{e}$, with μ fixed at the median empirical value of $0.7\times 10^{-8}$ (red). The shaded region shows the range of average pairwise nucleotide diversity ($\hat{\pi }$) for the species included in our analysis of $\hat{\pi }$ vs. evolvability and the darker region shows the range for species of conservation concern according to the IUCN Red List.

For traits under stabilizing selection at equilibrium, $V_{A}$ depends on both $N_{e}$ and mutation rate (μ) only when $N_{e}$ is very small and/or selection at quantitative trait loci is very weak, and converges to the neutral expectation of β=1 at the limit. As $N_{e}$ and/or the strength of selection increases, $V_{A}$ becomes independent of $N_{e}$ and only depends on μ (Fig. 4 and *SI Appendix*, Figs. S4 and S5). Consequently, the expected relationship between $V_{A}$ and π is likely weak if most of the variation in π is driven by differences in $N_{e}$ as opposed to differences in μ. Assuming most diversity is neutral, $\pi =4N_{e}\mu$, empirical estimates of $ln(N_{e})$ vary 2.8 times more than ln(μ) across multicellular eukaryotes (51, see also ref. 49). When these estimates are used to calculate predicted equilibrium values of $ln(V_{A})$ assuming an average selection coefficient of −0.001 (e.g. ref. 52), the resulting regression of $ln(V_{A})$ on ln(π) is not significantly different from zero, β=−0.053[−0.152−−0.025],P=0.200 (Fig. 4). Predictions under alternative parameterizations are shown in *SI Appendix*, Fig. S6. Under nonequilibrium conditions, stabilizing selection on a trait can speed up the rate at which $V_{A}$ equilibrates, further decoupling $V_{A}$ from π, although this effect is expected to be relatively weak for polygenic traits (*SI Appendix*, Fig. S8). The expected values of β are unchanged if $ln(V_{A})$ is substituted for $ln(I_{A})$, but not $h^{2}$.

## Discussion

**Fig. 5.**
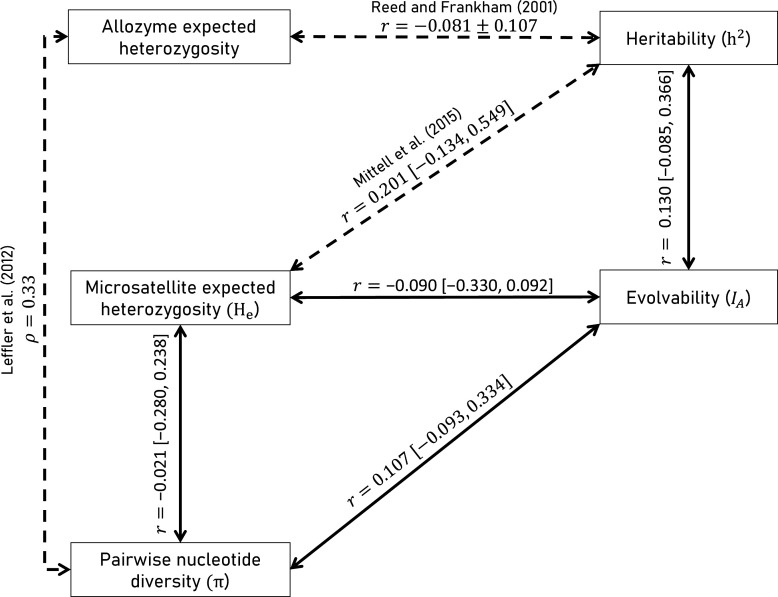
Interspecific correlations between measures of molecular genetic variation (*Left*) and quantitative genetic variation (*Right*) from analyses using data from over 20 species. Solid lines show estimates from this study and dotted lines show results reported in previous studies (14, 28, 29). Values show the Spearman rank (ρ) or Pearson (r) correlation coefficient ± the SE or 95% credible intervals in brackets. The correlation coefficient is not provided explicitly in ref. 14, so we calculated it directly from their published population-level model which excluded estimates from ref. 44. Note that ref. 28 report the correlation between allozyme diversity and *measured* average heritability, $\hat{h^{2}}$, and that the correlations reported between evolvability and both microsatellite and nucleotide diversity use log-transformed estimates. See ref. 33 for the estimated correlation between heritability and evolvability of individual traits.

Previous studies have shown that both microsatellite and allozyme diversity are poor predictors of heritability (14, 28). However, these findings may give little insight into whether conserving nucleotide diversity can maintain adaptive potential because these traditional markers are weakly correlated with nucleotide diversity (Figs. 3 and 5) and heritability itself may be an unreliable proxy. To assess whether contemporary molecular measures of genetic variation can predict adaptive potential, we estimated the strength of the relationship between nucleotide diversity and evolvability ($I_{A}$, mean-standardized $V_{A}$) across 108 species. Our results suggest that nucleotide diversity is a poor predictor of evolvability across species, with the best estimates showing only 1.1% of variation in $ln(I_{A})$ can be explained by variation in nucleotide diversity and that $I_{A}$ would only increase by 11.7% if nucleotide diversity were doubled. Similarly weak relationships were found across different measures of molecular and quantitative genetic variation (Fig. 5), suggesting that simple assays of molecular genetic diversity are unlikely to be useful for inferring a species’ adaptive potential.

A strong one-to-one relationship between $ln(V_{A})$ and ln(π) is only expected if quantitative traits are neutral. Our estimate of the slope is considerably less than one (β=0.160[−0.125−0.452]) which is instead consistent with quantitative traits being subject to stabilizing selection and weakly deleterious variants making a substantial contribution to existing $V_{A}$. This is somewhat at odds with classic conservation genetic theory, which assumes that effectively neutral genetic variation will underpin future adaptive responses and underlies guidelines such as the $N_{e}=500$ rule (8). However, under a trait-based model of adaptation, it is these currently deleterious variants that are likely to become advantageous when the optimal phenotype changes (10, 16, 53). The degree to which stabilizing selection can decouple the relationship between $ln(V_{A})$ and ln(π) depends critically on $N_{e}$, with theory predicting a poor association if most of the variation in π is driven by variation in $N_{e}$ (51, 54) unless $N_{e}$ is very low or selection is very weak (Fig. 4). Species of conservation concern might be expected to have lower $N_{e}$ and therefore a stronger $ln(V_{A})$-ln(π) relationship than the species surveyed here, yet our dataset spans a range of nucleotide diversities typical of such taxa (e.g., figure 1 in ref. 10) and shows no detectable relationship within this range. This indicates that the weak association between π and $I_{A}$ is not simply an artifact of sampling many nonthreatened species (11). Additionally, in using the mean $I_{A}$ of traits as a proxy for $V_{A}\left(w\right)$, we implicitly assume that traits are equally likely targets of future selection. It is probably more realistic that most future adaptive traits are those already closely associated with fitness and therefore under stronger selection than the average trait sampled here. If anything, this would further weaken the dependence of $V_{A}\left(w\right)$ on π.

It has been repeatedly suggested that departures from mutation–selection-drift equilibrium may decouple $V_{A}$ and π and contribute to a weak relationship between the two measures, even in very small populations where the relationship is otherwise expected to be strong (14, 45–47). We find that this idea is only partly true: under neutrality a population only has to come to equilibrium once, and in the generations thereafter the relationship between $ln(V_{A})$ and ln(π) will be close to one-to-one, irrespective of whether the population remains in equilibrium or experiences a change in population size. Conversely to previous suggestions (14, 47), this implies that the higher mutation rate of microsatellites may not offer any appreciable advantage over nucleotides in bottlenecked populations. Consistent with this, we find that microsatellite diversity is also weakly predictive of quantitative genetic variation, although this may be compounded by ascertainment bias for highly polymorphic microsatellite markers (21). In nonneutral models, $V_{A}$ reaches its equilibrium more quickly when the selection acting on the underlying loci is strong (55). As a result, $V_{A}$ can become uncoupled from nucleotide diversity measured at more weakly selected sites. With this in mind, we tested whether a metric which integrates information for putative selected diversity, $\pi_{N}/\pi_{S}$, would be a better predictor of $V_{A}$ than π since the loci contributing to $\pi_{N}$ should have selection coefficients more comparable to quantitative trait loci. While the association between the estimated ${\hat{\pi }}_{N}/{\hat{\pi }}_{S}$ and our measures of $V_{A}$ was too imprecise to draw firm conclusions, other considerations suggest the association is unlikely to be strong. First, $\pi_{N}/\pi_{S}$ is expected to depend on the mean strength of selection (and $N_{e}$) (43), yet there may be little variation in the mean strength of selection across species [although this has only been characterized in a few taxa (e.g. ref. 56)]. Second, the potential for selection to decouple $V_{A}$ from π declines as a trait becomes more polygenic, because selection on each individual locus then becomes very weak (55).

Given the substantial interspecific variation in trait evolvability (see also ref. 57), consistent with differences in $V_{A}\left(w\right)$ between populations and/or species (19), predicting this variation remains a priority. Future improvements to sampling and sequencing are unlikely to improve the predictive power of nucleotide diversity—we show that current estimates are already very accurate and only very modest gains in predictive power are expected from more precise estimates. Techniques for assessing other components of genetic health have moved toward more advanced predictions based on genomic annotation and conservation (e.g. refs. 58 and 59) and it is an open question whether newer functionally informed methods could also improve prediction of adaptive potential. However, it seems possible that any variants predicted to contribute to future adaptive responses may also be identified as being currently deleterious and contributing to the mutation load. If this were the case, the resulting trade-off between segregating load and adaptive potential may complicate conservation prioritization.

Adaptive potential represents only one component of population genetic health, and nucleotide diversity may still reflect other processes relevant to extinction risk. However, the evidence that nucleotide diversity predicts other genetic components of population genetic health, such as inbreeding and accumulation of deleterious mutations, remains mixed (10, 11). Reported associations between nucleotide diversity and IUCN Red List status also tend to be weak (10, 60), although this may partly reflect limitations of the Red List itself for indicating true extinction risk (11). Whether levels of $V_{A}$ of traits differ systematically among Red List categories remains unclear as estimates of $V_{A}$ are rarely available for species of conservation concern (*SI Appendix*, Table S9). This gap is particularly relevant given renewed discussion regarding inclusion of genetic data for Red List assessments (60, 61).

By compiling a large dataset of genetic variation across a diverse range of taxa and explicitly accounting for methodological variation among published estimates of evolvability, we show that nucleotide diversity fails to predict a species’ *true* level of quantitative genetic variation—the necessary component for a response to selection. This echoes earlier work that found weak relationships between other measures of molecular diversity and quantitative genetic variation (14, 28). Given our definition and proxy for adaptive potential (the capacity for an immediate response to directional selection following a change in environment), these results highlight that existing molecular genetic proxies are unsuitable for predicting the capacity of a species to adapt to changes in their environment. Longer-term responses will depend on both $N_{e}$ during the period of directional selection (62, 63) and new mutations (64). The degree to which π predicts this longer-term response depends on the accuracy with which π predicts future $N_{e}$, which may be poor in rapidly declining populations (65). Overall, the recent global emphasis on conserving adaptive potential is encouraging, but the absence of a widely accepted formal definition and reliance on weak proxies impedes effective population assessment and management.

## Materials and Methods

### Data.

2,040 new estimates of heritability ($h^{2}$) and 1,145 new estimates of evolvability ($I_{A}$, or the coefficient of additive genetic variance, $CV_{A}=\sqrt{I_{A}}$) were combined with 1,653 $h^{2}$ and 987 $I_{A}$ or $CV_{A}$ estimates from two previous meta-analyses (14, 66), producing a final dataset that included $h^{2}$ estimates from 473 publications representing 246 species, and $I_{A}$ estimates from 309 publications representing 193 species. Most new estimates were obtained by searching Web of Science for studies published between 1992 and 2022 in the journals “Journal of Evolutionary Biology,” “Evolution,” “Heredity,” and “Proceedings of the Royal Society B” with the topics “heritability” and “evolvability.” To maximize overlap with the molecular dataset, some new estimates were obtained with a targeted search across all journals using the Latin species name. If evolvability was not reported, we calculated it from its component parts where possible. For each estimate, we recorded trait type (condensed classification of 33: morphological, physiological, behavioral, life history, fitness), method of estimation (clonal, full/half-sib model, mid/single-parent–offspring regression, animal model, realized selection response), dimensional classification (linear, quadratic, cubic, meristic, time, other) and model structure (the number of fixed/random effects). Because evolvability estimates are not suitable for all traits (33, 67, 68) and often contain errors (69), we checked all published evolvability estimates for inclusion and discounted more than 700 erroneous estimates. We then kept only one estimate per-trait per-population (usually estimates from the largest sample) to avoid nonindependence.

Single- and multilocus estimates of autosomal gene diversity compiled by ref. 14 provided the basis for our molecular dataset. This included 2,788 microsatellite expected heterozygosity ($H_{e}$) estimates in 71 species and 1,777 pairwise nucleotide diversity (π) estimates at putatively neutral sites (silent, synonymous, fourfold degenerate, noncoding and intronic sites) in 45 species. To increase overlap between the molecular and quantitative genetic datasets, further π estimates were sourced from other meta-analyses (42, 49, 70–72) and by searching Web of Science with the keywords “genetic diversity” or “nucleotide diversity” and the Latin species name. We additionally collected genome-wide diversity estimates for 47 species from the Tree of life data portal (73). To obtain estimates of diversity at “functional” (presumed selected) sites, we revisited the original publications and recorded estimates of nonsynonymous or zerofold degenerate pairwise nucleotide diversity ($\pi_{N}$). For each population, a weighted-mean diversity was first calculated within publications, giving a total of 545 population-level microsatellite $\tilde{H_{e}}$ estimates (100 species), 496 $\tilde{\pi }$ estimates (192 species), and 172 ${\tilde{\pi }}_{N}$ estimates (88 species). Diversity was then averaged across publications to give the population mean $\hat{\pi }$ and $\hat{H_{e}}$ used in the main analyses. Averages were weighted to prioritize estimates based on more loci and from populations assayed for $h^{2}$ and $I_{A}$ (details in *SI Appendix*). The relevant data structures for the analyses of quantitative and molecular genetic variation are summarized in Table 1.

### Analyses.

All statistical analyses involved Bayesian linear mixed models fitted using the package MCMCglmm v2.37 (74) in R v4.4.2 (75). Scaled (by 1,000) $F_{1,1}$ priors were used for all random-effect variance components and an inverse gamma prior, with shape and scale equal to 0.002, was used for the residual variance component. Normal priors with zero mean and large variances ($10^{8}$) were used for the fixed effects. The MCMC chains were run for 500,000 iterations with a burn-in period of 100,000, sampling every 200 iterations. Significance was assessed using pMCMC values (76) or a Wald test for omnibus tests of multicategory factors where the posterior means and covariance matrix of the effects were used.

#### Nucleotide diversity vs. evolvability.

As theory predicts a proportional relationship between $V_{A}$ and genetic diversity (*SI Appendix*, section 3), we regressed $ln(\hat{I_{A}})$ on $ln(\hat{\pi })$ with regression coefficient β. We do not adjust for methodological heterogeneity among $\hat{\pi }$ estimates as this variation reflects the practical challenge of inferring $V_{A}$ from empirical data. True variation in $ln(I_{A})$ among species, after accounting for $ln(\hat{\pi })$, was estimated by fitting *phylogenetic* and *nonphylogenetic* species effects as random. The phylogenetic covariance structure was assumed to be proportional to the amount of time two species have shared ancestry, based on a phylogeny constructed from published divergence-time estimates (Fig. 1; 77). The tree was scaled to unit length such that the estimated variance in the phylogenetic ($V_{ln(I_{A}):\;\text{Phy}}$) and nonphylogenetic ($V_{ln(I_{A}):\;\text{S}}$) species effects sum to give the total remaining between-species variation in $ln(I_{A})$. Differences in $ln(I_{A})$ estimation were accounted for with fixed effects for *type of relative* and the number of *fixed* and *random effect terms*. Trait differences were modeled with fixed effects for *trait category* and *trait dimension* and a random effect for *trait* identity. A random *publication* effect was fitted to capture remaining differences among studies.

From the model, two measures of the relationship between $\hat{\pi }$ and $I_{A}$ were calculated. First, the coefficient of determination,

$$\begin{array}{c} R^{2}=\frac{\beta^{2}V_{ln(\hat{\pi })}}{\beta^{2}V_{ln(\hat{\pi })}+V_{ln(I_{A}):\;\text{Phy}}+V_{ln(I_{A}):\;\text{S}}}sgn\beta , \end{array}$$

where the denominator is the total between-species variance in $ln(I_{A})$ and $V_{ln(\hat{\pi })}$ is the variance in log-transformed nucleotide diversity estimates such that the product $\beta^{2}V_{ln(\hat{\pi })}$ is the variation in $ln(I_{A})$ owing to variation in $ln(\hat{\pi })$. The sign of the regression coefficient is carried to allow for negative $R^{2}$ values (as in ref. 14). As with $h^{2}$, $R^{2}$ is prone to a “rubber ruler” effect (67) as it is dependent on the amount of variation in $I_{A}$. This means that a high $R^{2}$ would only indicate that molecular measures are useful if there is sufficient variation in $I_{A}$ to expect meaningful differences in adaptive potential between species. Therefore, we also report $2^{\beta }$, the expected proportional change in mean $I_{A}$ if $\hat{\pi }$ is doubled.

In addition, we fitted an equivalent bivariate model with both $ln(\hat{I_{A}})$ and $ln(\hat{\pi })$ as response variables. This allowed us to include species for which quantitative genetic data was available but $\hat{\pi }$ was missing but available for a close relative. It also allowed us to control for any systematic differences in $\hat{\pi }$ due to differences in sampling/estimation methodology. See *SI Appendix* for details.

Nonsynonymous diversity ($\pi_{N}$) is strongly correlated with neutral (synonymous) genetic diversity (42) meaning that $\pi_{N}$ alone has limited scope to better predict evolvability (14). Instead, we used the ratio of nonsynonymous (or zerofold degenerate) and synonymous (or fourfold degenerate) diversity ($\pi_{N}/\pi_{S}$) as a measure of functional variation. The relationship between $ln(I_{A})$ and $ln({\hat{\pi }}_{N}/{\hat{\pi }}_{S})$ was assessed using the same univariate and bivariate model structures described above, with $ln({\hat{\pi }}_{N}/{\hat{\pi }}_{S})$ replacing $ln(\hat{\pi })$.

#### Microsatellite diversity vs. evolvability.

Before assessing the relationship between microsatellite diversity and evolvability, we first compared traditional and contemporary measures of genetic variation using two bivariate models. For molecular genetic variation, the responses were species-average $\hat{\pi }$ and species-average microsatellite $\hat{H_{e}}$, fitted across 57 species with available estimates of both measures. For the quantitative genetic measures, the responses were 1,976 estimates of $h^{2}$ and $I_{A}$ in 183 species for which both quantitative genetic measures were available. In the quantitative genetic model, *Species* was included as a random effect, allowing the (co)variance between $h^{2}$ and $I_{A}$ to be partitioned into among-species and among-traits (within-species) components. For both models, the correlation coefficient (r) was calculated from posterior samples of the (co)variance matrices; for the quantitative genetic model, correlations were estimated separately at the species- and trait-level. We then assessed the relationship between $ln(I_{A})$ and microsatellite $ln(\hat{H_{e}})$ using the same univariate and bivariate model structures described for the analyses of nucleotide diversity, with $ln(\hat{H_{e}})$ replacing $ln(\hat{\pi })$ as a predictor.

#### Precision of estimates.

The 496 estimates of $\tilde{\pi }$ (within-publication) across 192 species were analyzed using a simple linear mixed model with species and population effects as random. The simplest model assumed that the estimation errors were homogenous across publications/populations. The $R^{2}$ between $\hat{\pi }$ and true values (π and species-mean π, $\bar{\pi }$) depend on the variances of these effects/errors and also how many $\tilde{\pi }$ contribute to $\hat{\pi }$. The reported $R^{2}$ were obtained for the subset of $\hat{\pi }$ used in the main analyses. We also assessed the repeatability of nucleotide diversity under more complex error structures and after accounting for possible systematic differences between estimates, but the conclusions were broadly the same. See *SI Appendix* for details.

#### Theoretical expectations.

To explore the expected relationship between $V_{A}$ and genetic diversity, we derived the partial derivatives of $ln(V_{A})$ with respect to the logged mutation rate, μ, and effective population size, $N_{e}$, under a range of models for drift-selection-mutation, both at equilibrium and out of equilibrium (50, 55, 78, 79). From these we approximated the expected regression of $ln(V_{A})$ on $ln\left(\pi \right)\approx ln\left(4\right)+ln\left(\mu \right)+ln\left(N_{e}\right)$ as

$$\begin{array}{c} \beta \approx \frac{Var\left(ln\left(\mu \right)\right)\frac{\partial ln(V_{A})}{\partial ln(\mu )}+Var\left(ln\left(N_{e}\right)\right)\frac{\partial ln(V_{A})}{\partial ln(N_{e})}}{Var(ln(\pi ))} \end{array}$$

assuming that ln(μ) and $ln(N_{e})$ are not strongly correlated. If they are negatively correlated (49, 80) the regression will be shallower than this approximation predicts. As the regression depends on the relative contributions of variation in $ln(N_{e})$ and ln(μ) to variation in ln(π) across species, we used empirical estimates of $N_{e}$ and μ for 110 multicellular eukaryotic species (51) to generate predicted equilibrium values of $V_{A}$. We then fitted a simple linear model to these predicted values to illustrate the expected equilibrium relationship between $ln(V_{A})$ on ln(π) across species. All predicted values were calculated assuming 100 unlinked loci 1,000 base pairs in length (e.g. ref. 81). See *SI Appendix* for details.

## Supplementary Material

Appendix 01 (PDF)

## Data Availability

Data have been deposited in Github (https://github.com/katieabson/QvM26) (82)

## References

[1] F. W. Allendorf, Genetics and the conservation of natural populations: Allozymes to genomes. Mol. Ecol. **26**, 420–430 (2017). 27933683 10.1111/mec.13948

[2] F. W. Allendorf , Conservation and the Genomics of Populations (Oxford University Press, Oxford, New York, ed. 3, 2022).

[3] R. Frankham, Genetics and extinction. Biol. Conserv. **126**, 131–140 (2005).

[4] C. F. Olson-Manning, M. R. Wagner, T. Mitchell-Olds, Adaptive evolution: Evaluating empirical support for theoretical predictions. Nat. Rev. Genet. **13**, 867–877 (2012). 23154809 10.1038/nrg3322 PMC3748133

[5] CBD, Decision adopted by the conference of the parties to the convention on biological diversity 15/4. Kunming-Montreal Global Biodiversity Framework (2022). https://www.cbd.int/doc/decisions/cop-15/cop-15-dec-04-en.pdf.

[6] R. Lande, Mutation and conservation. Conserv. Biol. **9**, 782–791 (1995).

[7] I. R. Franklin, R. Frankham, How large must populations be to retain evolutionary potential? Anim. Conserv. Forum **1**, 69–70 (1998).

[8] I. Franklin, “Evolutionary changes in small populations” in *Conservation Biology, an Evolutionary-Ecological Perspective*, M. E. Soulé & B. A. Wilcox, Eds.(Sinauer Associates, Sunderland, Massachusetts, 1980), pp. 135–149.

[9] M. E. Soulé, “Thresholds for survival: Maintaining fitness and evolutionary potential” in *Conservation Biology, an Evolutionary-Ecological Perspective*, M. E. Soulé & B. A. Wilcox, Eds. (Sinauer Associates, 1980), pp. 151–170.

[10] J. C. Teixeira, C. D. Huber, The inflated significance of neutral genetic diversity in conservation genetics. Proc. Natl. Acad. Sci. U.S.A. **118**, e2015096118 (2021). 33608481 10.1073/pnas.2015096118 PMC7958437

[11] M. Kardos , The crucial role of genome-wide genetic variation in conservation. Proc. Natl. Acad. Sci. U.S.A. **118**, e2104642118 (2021). 34772759 10.1073/pnas.2104642118 PMC8640931

[12] A. García-Dorado, A. Caballero, Neutral genetic diversity as a useful tool for conservation biology. Conserv. Genet. **22**, 541–545 (2021).

[13] Y. Willi , Conservation genetics as a management tool: The five best-supported paradigms to assist the management of threatened species. Proc. Natl. Acad. Sci. U.S.A. **119**, e2105076119 (2022). 34930821 10.1073/pnas.2105076119 PMC8740573

[14] E. A. Mittell, S. Nakagawa, J. D. Hadfield, Are molecular markers useful predictors of adaptive potential? Ecol. Lett. **18**, 772–778 (2015). 25989024 10.1111/ele.12454

[15] R. G. Shaw, From the past to the future: Considering the value and limits of evolutionary prediction. Am. Nat. **193**, 1–10 (2019). 30624100 10.1086/700565

[16] R. A. Fisher, The Genetical Theory of Natural Selection (Clarendon Press, Oxford, 1930).

[17] G. R. Price, Fisher’s ‘fundamental theorem’ made clear. Ann. Hum. Genet. **36**, 129–140 (1972). 4656569 10.1111/j.1469-1809.1972.tb00764.x

[18] A. Burt, The evolution of fitness. Evolution **49**, 1–8 (1995). 28593672 10.1111/j.1558-5646.1995.tb05954.x

[19] T. Bonnet , Genetic variance in fitness indicates rapid contemporary adaptive evolution in wild animals. Science **376**, 1012–1016 (2022). 35617403 10.1126/science.abk0853

[20] A. P. Hendry, D. J. Schoen, M. E. Wolak, J. M. Reid, The contemporary evolution of fitness. Annu. Rev. Ecol. Evol. Syst. **49**, 457–476 (2018).

[21] P. de Villemereuil , Little adaptive potential in a threatened passerine bird. Curr. Biol. **29**, 889–894 (2019). 30799244 10.1016/j.cub.2019.01.072

[22] A. F. Agrawal, J. R. Stinchcombe, How much do genetic covariances alter the rate of adaptation? Proc. R. Soc. B Biol. Sci. **276**, 1183–1191 (2009). 10.1098/rspb.2008.1671 PMC2679087 19129097

[23] R. Lande, A quantitative genetic theory of life history evolution. Ecology **63**, 607–615 (1982).

[24] T. F. Hansen, D. Houle, Measuring and comparing evolvability and constraint in multivariate characters. J. Evol. Biol. **21**, 1201–1219 (2008). 18662244 10.1111/j.1420-9101.2008.01573.x

[25] D. S. Falconer, T. F. Mackay, Introduction To Quantitative Genetics (Longman, Harlow, UK, ed. 4, 1996).

[26] B. Charlesworth, D. Charlesworth, Elements of Evolutionary Genetics (Roberts and Company, Greenwood Village, 2010).

[27] J. Zeng , Signatures of negative selection in the genetic architecture of human complex traits. Nat. Genet. **50**, 746–753 (2018). 29662166 10.1038/s41588-018-0101-4

[28] D. H. Reed, R. Frankham, How closely correlated are molecular and quantitative measures of genetic variation? A meta-analysis. Evolution **55**, 1095–1103 (2001). 11475045 10.1111/j.0014-3820.2001.tb00629.x

[29] E. M. Leffler , Revisiting an old riddle: What determines genetic diversity levels within species?. PLoS Biol. **10**, e1001388 (2012). 22984349 10.1371/journal.pbio.1001388 PMC3439417

[30] Ü. Väli, A. Einarsson, L. Waits, H. Ellegren, To what extent do microsatellite markers reflect genome-wide genetic diversity in natural populations? Mol. Ecol. **17**, 3808–3817 (2008). 18647238 10.1111/j.1365-294X.2008.03876.x

[31] M. C. Fischer , Estimating genomic diversity and population differentiation - an empirical comparison of microsatellite and SNP variation in *Arabidopsis halleri*. BMC Genom. **18**, 69 (2017). 10.1186/s12864-016-3459-7 PMC5225627 28077077

[32] J. Pérez-González , Comparative analysis of microsatellite and SNP markers for genetic management of red deer. Animals **13**, 3374 (2023). 37958129 10.3390/ani13213374 PMC10650148

[33] T. F. Hansen, C. Pélabon, D. Houle, Heritability is not evolvability. Evol. Biol. **38**, 258–277 (2011).

[34] D. Houle, Comparing evolvability and variability of quantitative traits. Genetics **130**, 195–204 (1992). 1732160 10.1093/genetics/130.1.195 PMC1204793

[35] J. L. A. Wood, M. C. Yates, D. J. Fraser, Are heritability and selection related to population size in nature? Meta-analysis and conservation implications. Evol. Appl. **9**, 640–657 (2016). 27247616 10.1111/eva.12375 PMC4869407

[36] T. F. Hansen, C. Pélabon, Evolvability: A quantitative-genetics perspective. Annu. Rev. Ecol. Evol. Syst. **52**, 153–175 (2021).

[37] A. A. Hoffmann, J. Merilä, T. N. Kristensen, Heritability and evolvability of fitness and nonfitness traits: Lessons from livestock. Evolution **70**, 1770–1779 (2016). 27346243 10.1111/evo.12992

[38] C. Pélabon , Evolvability: Progress and key questions. BioScience **75**, 1042–1057 (2025). 41367904 10.1093/biosci/biaf111 PMC12683529

[39] G. Yu, Using ggtree to visualize data on tree-like structures. Curr. Protoc. Bioinf. **69**, e96 (2020). 10.1002/cpbi.96 32162851

[40] W. Gearty, L. A. Jones, Rphylopic: An R package for fetching, transforming, and visualising PhyloPic silhouettes. Methods Ecol. Evol. **14**, 2700–2708 (2023).

[41] IUCN, The IUCN Red List of Threatened Species. Version 2025-1 (2025). https://www.iucnredlist.org (Accessed 13 August 2025).

[42] J. Romiguier , Comparative population genomics in animals uncovers the determinants of genetic diversity. Nature **515**, 261–263 (2014). 25141177 10.1038/nature13685

[43] J. J. Welch, A. Eyre-Walker, D. Waxman, Divergence and polymorphism under the nearly neutral theory of molecular evolution. J. Mol. Evol. **67**, 418–426 (2008). 18818860 10.1007/s00239-008-9146-9

[44] V. L. Corre, Variation at two flowering time genes within and among populations of *Arabidopsis thaliana*: Comparison with markers and traits. Mol. Ecol. **14**, 4181–4192 (2005). 16262868 10.1111/j.1365-294X.2005.02722.x

[45] R. Lande, G. F. Barrowclough, “Effective population size, genetic variation, and their use in population management” in *Viable Populations for Conservation*, M. E. Soulé, Ed. (Cambridge University Press, Cambridge, 1987), pp. 87–124.

[46] M. Lynch, “A quantitative-genetic perspective on conservation issues” in *Conservation Genetics: Case Histories From Nature*, J. C. Avise, J. L. Hamrick, Eds. (Chapman and Hall, New York, 1996), pp. 471–501.

[47] E. G. Boulding, “Genetic diversity, adaptive potential, and population viability in changing environments” in *Conservation Biology: Evolution in Action*, S. P. Carroll, C. W. Fox, Eds. (Oxford University Press, Oxford, 2008), pp. 199–219.

[48] D. Houle, B. Morikawa, M. Lynch, Comparing mutational variabilities. Genetics **143**, 1467–1483 (1996). 8807316 10.1093/genetics/143.3.1467 PMC1207413

[49] Y. Wang, D. J. Obbard, Experimental estimates of germline mutation rate in eukaryotes: A phylogenetic meta-analysis. Evol. Lett. **7**, 216–226 (2023). 37475753 10.1093/evlett/qrad027 PMC10355183

[50] R. Bürger, G. P. Wagner, F. Stettinger, How much heritable variation can be maintained in finite populations by mutation-selection balance? Evolution **43**, 1748–1766 (1989). 28564325 10.1111/j.1558-5646.1989.tb02624.x

[51] L. Lewin, A. Eyre-Walker, A comparative analysis of long-term effective population sizes across eukaryotes. Mol. Ecol. **35**, e70265 (2026). 41674470 10.1111/mec.70265 PMC12895298

[52] J. Zeng , Widespread signatures of natural selection across human complex traits and functional genomic categories. Nat. Commun. **12**, 1164 (2021). 33608517 10.1038/s41467-021-21446-3 PMC7896067

[53] R. Frankham, C. J. A. Bradshaw, B. W. Brook, Genetics in conservation management: Revised recommendations for the 50/500 rules, Red List criteria and population viability analyses. Biol. Conserv. **170**, 56–63 (2014).

[54] M. Lynch , Genetic drift, selection and the evolution of the mutation rate. Nat. Rev. Genet. **17**, 704–714 (2016). 27739533 10.1038/nrg.2016.104

[55] C. Chevalet, An approximate theory of selection assuming a finite number of quantitative trait loci. Genet. Sel. Evol. **26**, 379 (1994).

[56] D. Castellano, M. C. Macià, P. Tataru, T. Bataillon, K. Munch, Comparison of the full distribution of fitness effects of new amino acid mutations across great apes. Genetics **213**, 953–966 (2019). 31488516 10.1534/genetics.119.302494 PMC6827385

[57] L. Zijmers, K. L. Abson, J. D. Hadfield, A. Eyre-Walker, Levels of additive genetic variation vary substantially between species. bioRxiv (2026). 10.64898/2026.01.22.701036 (Accessed 23 January 2026). PMC13278422 42313750

[58] C. Bozzuto, I. Biebach, S. Muff, A. R. Ives, L. F. Keller, Inbreeding reduces long-term growth of Alpine ibex populations. Nat. Ecol. Evol. **3**, 1359–1364 (2019). 31477848 10.1038/s41559-019-0968-1

[59] C. Grossen, F. Guillaume, L. F. Keller, D. Croll, Purging of highly deleterious mutations through severe bottlenecks in Alpine ibex. Nat. Commun. **11**, 1001 (2020). 32081890 10.1038/s41467-020-14803-1 PMC7035315

[60] C. Schmidt, S. Hoban, M. Hunter, I. Paz-Vinas, C. J. Garroway, Genetic diversity and IUCN Red List status. Conserv. Biol. **37**, e14064 (2023). 36751982 10.1111/cobi.14064

[61] C. M. McLaughlin, C. Hinshaw, S. Sandoval-Arango, M. Zavala-Paez, J. A. Hamilton, Redlisting genetics: Towards inclusion of genetic data in IUCN Red List assessments. Conserv. Genet. **26**, 213–223 (2025).

[62] A. Robertson, A theory of limits in artificial selection. Proc. R. Soc. B Biol. Sci. **153**, 234–249 (1960).

[63] K. Weber, Increased selection response in larger populations. I. Selection for wing-tip height in drosophila melanogaster at three population sizes. Genetics **125**, 579 (1990). 2116358 10.1093/genetics/125.3.579 PMC1204084

[64] W. G. Hill, Rates of change in quantitative traits from fixation of new mutations. Proc. Natl. Acad. Sci. U.S.A. **79**, 142–145 (1982). 6948296 10.1073/pnas.79.1.142 PMC345678

[65] M. Exposito-Alonso, Signals of consistent genetic diversity decline are not yet measurable in global meta-analysis. bioRxiv [Preprint] (2025). 10.1101/2025.08.01.663988 (Accessed 10 October 2025).

[66] E. A. Young, E. Postma, Low interspecific variation and no phylogenetic signal in additive genetic variance in wild bird and mammal populations. Ecol. Evol. **13**, e10693 (2023). 37933323 10.1002/ece3.10693 PMC10625858

[67] D. Houle, C. Pélabon, G. P. Wagner, T. F. Hansen, Measurement and meaning in biology. Q. Rev. Biol. **86**, 3–34 (2011). 21495498 10.1086/658408

[68] C. Pélabon, C. H. Hilde, S. Einum, M. Gamelon, On the use of the coefficient of variation to quantify and compare trait variation. Evol. Lett. **4**, 180–188 (2020). 32547779 10.1002/evl3.171 PMC7293077

[69] F. Garcia-Gonzalez, L. W. Simmons, J. L. Tomkins, J. S. Kotiaho, J. P. Evans, Comparing evolvabilities: Common errors surrounding the calculation and use of coefficients of additive genetic variation. Evolution **66**, 2341–2349 (2012). 22834736 10.1111/j.1558-5646.2011.01565.x

[70] V. Buffalo, Quantifying the relationship between genetic diversity and population size suggests natural selection cannot explain Lewontin’s Paradox. eLife **10**, e67509 (2021). 34409937 10.7554/eLife.67509 PMC8486380

[71] L. A. Bergeron , Evolution of the germline mutation rate across vertebrates. Nature **615**, 285–291 (2023). 36859541 10.1038/s41586-023-05752-y PMC9995274

[72] J. Chen, S. Glémin, M. Lascoux, Genetic diversity and the efficacy of purifying selection across plant and animal species. Mol. Biol. Evol. **34**, 1417–1428 (2017). 28333215 10.1093/molbev/msx088

[73] , Sequence locally, think globally: The Darwin Tree of Life Project. Proc. Natl. Acad. Sci. U.S.A. **119**, e2115642118 (2022). 35042805 10.1073/pnas.2115642118 PMC8797607

[74] J. D. Hadfield, MCMC methods for multi-response generalized linear mixed models: The MCMCglmm R package. J. Stat. Softw. **33**, 1–22 (2010).20808728

[75] R Core Team, R: A Language and Environment for Statistical Computing (R Foundation for Statistical Computing, Vienna, Austria, 2023).

[76] R. H. Baayen, D. J. Davidson, D. M. Bates, Mixed-effects modeling with crossed random effects for subjects and items. J. Mem. Lang. **59**, 390–412 (2008).

[77] S. Kumar , TimeTree 5: An expanded resource for species divergence times. Mol. Biol. Evol. **39**, msac174 (2022). 35932227 10.1093/molbev/msac174 PMC9400175

[78] G. Clayton, A. Robertson, Mutation and quantitative variation. Am. Nat. **89**, 151–158 (1955).

[79] B. D. H. Latter, Selection in finite populations with multiple alleles. II. Centripetal selection, mutation, and isoallelic variation. Genetics **66**, 165–186 (1970). 5512470 10.1093/genetics/66.1.165 PMC1212482

[80] M. Lynch , The divergence of mutation rates and spectra across the Tree of Life. EMBO Rep. **24**, EMBR202357561 (2023). 10.15252/embr.202357561 PMC10561183 37615267

[81] L. Xu , Average gene length is highly conserved in prokaryotes and eukaryotes and diverges only between the two kingdoms. Mol. Biol. Evol. **23**, 1107–1108 (2006). 16611645 10.1093/molbev/msk019

[82] K. L. Abson, QvM26. GitHub. https://github.com/katieabson/QvM26. Deposited 16 April 2026.

