# Supplementary material for: Nucleotide diversity is a poor predictor of short-term adaptive potential
Source: Proc Natl Acad Sci U S A. 2026 Jun 29;123(27):e2536181123. doi: 10.1073/pnas.2536181123 (PMC13342847; doi:10.1073/pnas.2536181123)
Supplement: Supplementary file 1 — Appendix 01 (PDF) [file pnas.2536181123.sapp.pdf]

PNAS

Supporting Information for

Nucleotide diversity is a poor predictor of short-term adaptive potential

Katie L. Abson, Lillith Zijmers, Elizabeth A. Mittell, Euan A. Young, Erik Postma, Adam Eyre-Walker, Jarrod D. Hadfield

Corresponding Author: Katie L. Abson.
E-mail: K.L.Abson@sms.ed.ac.uk

This PDF file includes:

Supporting text
Figs. S1 to S8
Tables S1 to S15
SI References

Supporting Information Text

Supplementary Methods and Results

1. Data

Quantitative genetic variation. Fewer estimates of evolvability were available compared with heritability since it is less frequently reported and because evolvability is not suitable for all traits (1–3). For many traits where evolvability is suitable yet was not reported, we were able to calculate it using the formula $I_A = V_A/\bar{X}^2$, where V_A is the additive genetic variance and $\bar{X}$ is the trait mean (note that only the denominator is squared). If neither V_A or the trait mean were reported, an evolvability estimate was still able to be calculated if an estimate of both the heritability, h^2 and the coefficient of variation $CV = \sqrt{V_P}/\bar{X}$, where V_P is the phenotypic variance, were available. Estimates included in the dataset are predominantly for wild populations, though some cultivated or lab-based populations were included.

For each estimate, we recorded additional information relating to the choice of trait and methodology. We recorded the trait type using a condensed classification of 2: morphological, physiological, behavioural, life history and fitness (lifetime breeding success), as both heritability and evolvability have been shown to vary among these categories (2, 4–6). Since evolvabilities depend on the dimension of a trait (2), we further classified estimates as: linear, quadratic, cubic, meristic, time and other. To deal with methodological differences, we recorded the method of estimation: clonal, full-sib, half-sib, mid/single-parent-offspring regression, animal model and realised selection response, since estimation based on different types of relatives or realised changes in the trait vary in the extent to which V_A is biased (7). Finally we recorded information relating to model structure (the number of fixed/random effects), as this can affect how much trait variation is estimated and partitioned among additive versus non-additive components (8).

Error in estimates of I_A and implications for the estimated relationship between $\ln(I_A)$ and $ln(\hat{\pi})$. We quantify the relationship between evolvability and nucleotide diversity using two measures calculated using the slope (β) of the regression of $ln(I_A)$ on $ln(\hat{\pi})$. First, R^2, which is the proportion of true between-species variance in $ln(I_A)$ explained by variance in $ln(\hat{\pi})$. Second, 2^β, which is the expected proportional increase in I_A if $\hat{\pi}$ were doubled. Whereas R^2 depends on the estimated between-species variance in $ln(I_A)$, 2^β only depends on the estimated slope.

In regression analyses, non-systematic (random) error in the response does not result in a biased slope, but the residual error is inflated. Since we do not include the residual variance when calculating the R^2, this measure is also unaffected by random error because $V_{ln(I_A):Phy}$ (phylogenetic between-species variance) and $V_{ln(I_A):S}$ (non-phylogenetic between species variance) are not biased by non-systematic errors in the estimates. In other words, we measure the R^2 of $ln(\overline{I_A})$ versus $ln(\hat{\pi})$, not $ln(\widehat{I_A})$ versus $ln(\hat{\pi})$. In contrast, systematic (non-random) errors can result in biased slopes and R^2 values. If the errors in I_A are correlated with $\hat{\pi}$, then the estimated slope can be biased upwards or downwards depending on the sign of the correlation. However, it is hard to imagine why such a correlation would exist and so we believe the estimated slope is likely unbiased. If the sign and magnitude of error varies over species, but is uncorrelated with $\hat{\pi}$, then $V_{ln(I_A):S}$ will be inflated, leading to a downward bias in the R^2. To minimise systematic bias, we discarded more than 700 published I_A estimates with identifiable errors (e.g., reported for unsuitable traits or calculation errors were made). We tried to account for remaining systematic bias in our analyses by including differences in estimation method and trait choice and by fitting random publication effects in our models. We therefore believe these effects of error in $\widehat{I_A}$ should be minimal.

Error in estimates of I_A downwardly bias estimates of $ln(I_A)$. One remaining source of systematic bias is that while estimates of I_A may be approximately unbiased on the arithmetic scale, on average they will be downwardly biased on the log-scale (the scale at which we analyse them). The downward bias arises because of Jensen's inequality (the log transform is concave) and will increase as the estimation error on I_A increases (9). This does not affect β unless precision in $\widehat{I_A}$ is correlated with $\hat{\pi}$, which again seems unlikely. If the precision of I_A estimates systematically varies among species after accounting for the terms described above, $V_{ln(I_A):S}$ may be inflated and the R^2 reduced. Approximate methods exist for accounting for this bias in estimates obtained from non-hierarchical models (9, 10). However, extending these methods to estimates from hierarchical models seems difficult, even if standard errors on variance estimates are given, plus a large minority (26%) of our I_A estimates did not come with an associated standard error or alternative measure of precision. Therefore, instead of directly accounting for this source of bias, below we approximate the extent to which systematic bias arising from log-transformation of the response is likely to affect our estimate of the R^2.

The expectation of the log of an estimated variance component, $ln(\hat{V})$, can be approximated using a second-order Taylor approximation as

$$E[ln(\hat{V})] = ln(E[\hat{V}]) - \frac{Var(\hat{V})}{2E[\hat{V}]^2} \qquad [1]$$

In simple non-hierarchical models with Gaussian response, the residual is the only estimated variance and its sampling distribution is a scaled-χ^2 with scale equal to the true variance divided by the degrees of freedom (V/ν). The mean and variance of this sampling distribution are V and $2V^2/\nu$ respectively, giving

$$E[ln(\hat{V})] = ln(V) - \frac{2V^2/\nu}{2V^2} = ln(V) - 1/\nu \qquad [2]$$

The bias is $-1/\nu$, as given in (9) by a different method. The coefficient of variation, $E[ln(\widehat{CV})]$, will have approximately the same bias since error in the mean is likely small compared to error in V (see 10 where this assumption is relaxed).

In hierarchical models the sampling distribution of the variances are not known, although for simple ANOVA-based approaches the first and second moments are known (11) (see 12 for corrections). For a model only containing family and residual effects $E[\widehat{V_A}] = V_A$, but $Var(\widehat{V_A})$ depends in a complicated way on V_A, the residual variance and the experimental design. Consequently, the bias requires knowledge of the unknown true heritability (h^2) even when the standard errors squared ($Var(\widehat{V_A})$) are available. For a balanced design the bias is

$$-\frac{Var(\widehat{V_A})}{2E[\widehat{V_A}]^2} = -\frac{Var(\widehat{V_A})}{2V_A^2} = -\frac{1}{n^2(c-1)}\left[\frac{N-1}{N-c}\left(\frac{1-h}{h}+1-r\right)^2 + 2nr\left(\frac{1-h}{h}+1-r\right) + n^2r^2\right] \quad [3]$$

where c is the number of families, n is the number of individuals per family and r is the relatedness of those individuals. $N = nc$. The variance in the bias across the 190 I_A estimates for which n, c and r (1 for clones, 1/2 for full-sibs, 1/4 for half-sibs) were recorded was 0.0011, assuming a true heritability of 0.38 (the mean estimated heritability). Given this is orders of magnitude lower than the estimated between-species variance in $ln(I_A)$, the systematic bias arising from log-transformation of the response is likely to have a negligible impact on our estimate of the R^2 between $ln(I_A)$ and $ln(\hat{\pi})$.

Nucleotide diversity. We prioritised estimates of pairwise nucleotide diversity (π) since this is the most widely reported measure of nucleotide variation. Where available, we recorded the number of individuals, loci, and sites over which the diversity estimate was based on, together with the level of sampling (few targeted loci or putatively genome-wide representative) and whether individuals from different populations had been pooled.

Calculating π estimates ($\tilde{\pi}$) used in the main analysis. For the 55 studies in 5 which reported nucleotide diversity, most estimates were obtained for the lowest level of sampling (often single loci). The median number of loci per publication/population was relatively modest (9 loci), with few estimates considered to be genome-wide representative (0.05%). Since these data were collected, studies that estimate genome-wide genetic diversity (i.e., based on whole-genomes or a subset obtained by reduced representation sequencing) have become more commonplace. However, locus-level diversities are usually not reported in these studies and the number of loci (as opposed to sites) is hard to define. In order to accommodate both types of data, we obtain an average estimate of diversity for each publication/species/population combination, which we call $\tilde{\pi}$. For the 43 studies which report genome-wide estimates, we use a simple average over the independent estimates for each population. For the other 67 studies which report estimates based on few targeted (groups of) loci, we form an average where each locus is given equal weight. For the main analyses in the paper we obtained genetic diversity estimates for each population ($\hat{\pi}$) by taking a (weighted) average of the publication-level estimates ($\tilde{\pi}$) for that species. For population m of species i:

$$\hat{\pi}_{im} = \sum_{j}^{n_i^{(p)}} \sum_{k}^{n_{ij}^{(e)}} w_{ijk}\tilde{\pi}_{ijk} \quad [4]$$

where $n_i^{(p)}$ is the number of populations of species i for which estimates exist and $n_{ij}^{(e)}$ is the number of studies reporting estimates for population j of species i. w is a weight with $\sum_j^{n_i^{(p)}} \sum_k^{n_{ij}^{(e)}} w_{ijk} = 1$. We chose a weighting scheme that would improve precision over a standard unweighted average. If a species had at least one genome-wide estimate of $\tilde{\pi}$ we took the unweighted average of all genome-wide estimates: the weight for an estimate is then zero if it is not genome-wide and $1/n_i^{(g)}$ otherwise, where $n_i^{(g)}$ is the number of genome-wide estimates for species i. If a species did not have any genome-wide estimates the weights were $n_{ijk}^{(l)}/N_i^{(l)}$ where $n_{ijk}^{(l)}$ is the number of loci sampled for that estimate, and $N_i^{(l)}$ is the aggregate number of loci sampled for that species: $N_i^{(l)} = \sum_j^{n_i^{(p)}} \sum_k^{n_{ij}^{(e)}} n_{ijk}^{(l)}$. In the few cases (5.6% of species) where we had estimates of π for the same population in which I_A was measured we restricted ourselves to estimates from that population. In such cases w_{ijk} is effectively set to zero whenever $j \neq m$ and the normalising constants described above were recalculated.

Assessing error in $\hat{\pi}$ by modelling the publication/population level estimates ($\tilde{\pi}$). In order to quantify how well our *estimate* of a population's genetic diversity ($\hat{\pi}_{im}$) predicts the true value (π_{im}) or the true species average over populations ($\overline{\pi}_i$) we performed a set of 'diversity analyses' on our publication-level estimates ($\tilde{\pi}$). A linear model of the form

$$\tilde{\pi}_{ijk} = c + (\Delta\overline{\pi})_i + (\Delta\pi)_{ij} + (\Delta\tilde{\pi})_{ijk} \quad [5]$$

was fitted, where c is the average diversity across all species, $\Delta\overline{\pi}_i$ is the deviation of species i from the global mean, $(\Delta\pi)_{ij}$ is the deviation of population j from the species mean and $(\Delta\tilde{\pi})_{ijk}$ is the estimation error. We fitted species and population deviations as random effects to estimate the between-species (σ_s^2) and between-species within-population (σ_p^2) variation in $\tilde{\pi}$, respectively. In the simplest model (reported in the main text) we assumed the error variance was constant, and a single error variance was estimated (σ_e^2). However, the error variance might be expected to vary over estimates in predictable ways. Because of this we also fitted models where the error variance of genome-wide estimates ($\sigma^2_{e^{(g)}}$) was allowed to be different

from the error variance of estimates obtained using a targeted set of loci. For these loci, the most complicated model was allowed to depend on the aggregate number of loci sampled in that study ($n^{(l)}$):

$$\sigma^2_{e_{ijk}} = \sigma^2_{e(0)} + \frac{1}{n^{(l)}_{ijk}}\sigma^2_{e(1)} \quad [6]$$

As the number of loci becomes large the error variance tends to $\sigma^2_{e(0)}$ and we may expect this to be roughly comparable to $\sigma^2_{e(g)}$. It should be noted that the definition of population is somewhat nebulous and inconsistent across studies - in some cases populations may be defined as set of individuals in a very restricted area, yet for others studies, a population may constitute individuals over a much broader range, such as a country. If the definition of a population is too broad, the estimate of diversity will be inflated by $1/(1 - F_{ST})$ and its variance will be reduced since some of the between-population variance in π will be partly averaged over. Many studies (49 out of 110) reported estimates where individuals were explicitly 'pooled' from multiple populations. Consequently, we also fitted analyses where two separate values for $\sigma^2_{e(g)}$ and two separate values for $\sigma^2_{e(0)}$ were fitted, depending on whether the estimates were pooled or not.

In addition to different estimates having different variances they may also have different means. As discussed, pooled estimates may be expected to have higher π estimates due to population structure. The Tree of life (ToL) data portal (13) was an important source of new whole-genome diversity estimates for our study. However, these estimates are derived from a k-mer distribution (14) of a single genome per species. It has been suggested that k-mer based estimates may give higher estimates of diversity than standard approaches since they are better able to pick up nonreference variation (15). In order to accommodate these potential systematic differences we reran our five error models with 'pooled', 'non-pooled k-mer' and 'non-pooled standard' effects fitted as fixed.

The models fitted to all available diversity estimates (496 $\tilde{\pi}$ estimates from 469 populations of 192 species) irrespective of whether they were from species that had estimates of I_A or h^2 or not. Prior and MCMC specifications followed those for other analyses (see Section 2). The parameter estimates are summarised in Tables S1 and S2 which do, and do not, control for systematic differences, respectively. While the point estimates for such simple models are likely to have desirable properties (16, 17) credible intervals should be treated with caution - although the response was heavily right-skewed, it was treated as Gaussian in order to obtain variance estimates on the right scale. Estimates of $\tilde{\pi}$ based on samples of pooled populations were significantly larger (0.003 [0.002 – 0.005], P<0.5×10^{-3}) than those based on unpooled samples, as were k-mer based estimates (0.007 [0.004 – 0.009], P<0.5×10^{-3}). These values were obtained from the simplest model, but are consistent with the results of the models with more complex error structures.

Table S1. Diversity analyses variance estimates (scaled by 10^4) for models that did not control for systematic effects. Models A–E were fitted to the publication/population-level diversity estimates ($\tilde{\pi}$). In all models the the between-species (σ^2_p) and between-population (σ^2_s) variances in π were estimated. In the most complicated model (Model E) the error variance of genome-wide estimates was allowed to be different between pooled samples ($\sigma^2_{e(g|p)}$) and non-pooled ($\sigma^2_{e(g|np)}$) samples. For non-genome wide estimates the error variance when the number of loci is infinite is $\sigma^2_{e(0|p)}$ for pooled samples and $\sigma^2_{e(0|np)}$ for non-pooled. For non-genome wide estimates the error variance scales with $1/n^{(l)}$ by a factor $\sigma^2_{e(1)}$, where $n^{(l)}$ is the number of loci used to make the estimate. Models A-C assumed pooled and non-pooled samples are equivalent. Model A assumed genome-wide and non-genome-wide estimates are equivalent. Models A, B and D assume $\sigma^2_{e(1)} = 0$. $\overline{\sigma^2_e}$ is the average (over species) residual variance of $\tilde{\pi}$ and $\overline{\sigma^2_\epsilon}$ is the average residual variance of $\hat{\pi}$ (see below).

	Model A	Model B	Model C	Model D	Model E
σ^2_s	1.166[0.913 – 1.433]	1.188[0.953 – 1.482]	1.192[0.905 – 1.457]	1.190[0.937 – 1.459]	1.199[0.947 – 1.487]
σ^2_p	0.072[0.024 – 0.119]	0.077[0.023 – 0.120]	0.078[0.030 – 0.122]	0.100[0.053 – 0.141]	0.095[0.053 – 0.134]
$\sigma^2_{e(g\|p)}$	0.105[0.064 – 0.152]	0.076[0.037 – 0.127]	0.075[0.037 – 0.124]	0.014[0.000 – 0.104]	0.015[0.000 – 0.102]
$\sigma^2_{e(g\|np)}$	0.105[0.064 – 0.152]	0.076[0.037 – 0.127]	0.075[0.037 – 0.124]	0.064[0.029 – 0.110]	0.066[0.030 – 0.111]
$\sigma^2_{e(0\|p)}$	0.105[0.064 – 0.152]	0.128[0.075 – 0.191]	0.100[0.043 – 0.168]	0.036[0.000 – 0.149]	0.011[0.000 – 0.099]
$\sigma^2_{e(0\|np)}$	0.105[0.064 – 0.152]	0.128[0.075 – 0.191]	0.100[0.043 – 0.168]	0.119[0.072 – 0.179]	0.091[0.036 – 0.149]
$\sigma^2_{e(1)}$	0.000[0.000 – 0.000]	0.000[0.000 – 0.000]	0.095[0.000 – 0.401]	0.000[0.000 – 0.000]	0.146[0.000 – 0.450]
$\overline{\sigma^2_e}$	0.105[0.064 – 0.152]	0.085[0.049 – 0.137]	0.084[0.050 – 0.132]	0.059[0.030 – 0.103]	0.063[0.032 – 0.102]
$\overline{\sigma^2_\epsilon}$	0.075[0.046 – 0.109]	0.060[0.035 – 0.097]	0.059[0.035 – 0.094]	0.041[0.020 – 0.073]	0.043[0.021 – 0.071]

Determining how error in $\hat{\pi}$ biases the estimated relationship between $ln(\pi)$ and $ln(I_A)$. Imagine the relationship between $E[I_A]$ and π is linear and causal with regression coefficient β:

$$E[I_A]_{im} = \beta\pi_{im} + \delta_{im} \quad [7]$$

where δ_{im} is the residual. When fitting the regression, however, we replace π_{im} with $\hat{\pi}_{im}$. $\hat{\pi}_{im}$ will deviate from π_{im} for two reasons. First, population differences in π ($\Delta\pi$) will cause a deviation if estimates from populations other than m are used to obtain $\hat{\pi}_{im}$. These deviations are Berkson in nature and will not result in biased estimates of β (18). Second, estimation errors

Table S2. Diversity analyses variance estimates (scaled by 10^4) for models that controlled for systematic differences between ‘pooled’, ‘non-pooled k-mer’ and ‘non-pooled standard’ estimates. Models A–E were fitted to the publication/population-level diversity estimates ($\tilde{\pi}$). In all models the the between-species (σ^2_p) and between-population (σ^2_s) variances in π were estimated. In the most complicated model (Model E) the error variance of genome-wide estimates was allowed to be different between pooled samples ($\sigma^2_{e(g|p)}$) and non-pooled ($\sigma^2_{e(g|np)}$) samples. For non-genome wide estimates the error variance when the number of loci is infinite is $\sigma^2_{e(0|p)}$ for pooled samples and $\sigma^2_{e(0|np)}$ for non-pooled. For non-genome wide estimates the error variance scales with $1/n^{(l)}$ by a factor $\sigma^2_{e(1)}$, where $n^{(l)}$ is the number of loci used to make the estimate. Models A-C assumed pooled and non-pooled samples are equivalent. Model A assumed genome-wide and non-genome-wide estimates are equivalent. Models A, B and D assume $\sigma^2_{e(1)} = 0$. $\overline{\sigma^2_e}$ is the average (over species) residual variance of $\tilde{\pi}$ and $\overline{\sigma^2_\epsilon}$ is the average residual variance of $\hat{\pi}$ (see below).

	Model A	Model B	Model C	Model D	Model E
σ^2_s	1.041[0.808 – 1.271]	1.054[0.849 – 1.312]	1.053[0.831 – 1.310]	1.054[0.830 – 1.323]	1.065[0.814 – 1.318]
σ^2_p	0.074[0.032 – 0.117]	0.104[0.057 – 0.146]	0.105[0.051 – 0.141]	0.108[0.066 – 0.144]	0.107[0.067 – 0.146]
$\sigma^2_{e(g\|p)}$	0.094[0.060 – 0.139]	0.025[0.001 – 0.069]	0.025[0.001 – 0.070]	0.023[0.000 – 0.159]	0.021[0.000 – 0.146]
$\sigma^2_{e(g\|np)}$	0.094[0.060 – 0.139]	0.025[0.001 – 0.069]	0.025[0.001 – 0.070]	0.021[0.001 – 0.058]	0.022[0.001 – 0.061]
$\sigma^2_{e(0\|p)}$	0.094[0.060 – 0.139]	0.112[0.069 – 0.162]	0.099[0.049 – 0.154]	0.095[0.001 – 0.207]	0.079[0.001 – 0.190]
$\sigma^2_{e(0\|np)}$	0.094[0.060 – 0.139]	0.112[0.069 – 0.162]	0.099[0.049 – 0.154]	0.112[0.065 – 0.164]	0.095[0.050 – 0.156]
$\sigma^2_{e(1)}$	0.000[0.000 – 0.000]	0.000[0.000 – 0.000]	0.048[0.000 – 0.265]	0.000[0.000 – 0.000]	0.057[0.000 – 0.300]
$\overline{\sigma^2_e}$	0.094[0.060 – 0.139]	0.040[0.016 – 0.082]	0.041[0.017 – 0.083]	0.040[0.016 – 0.079]	0.040[0.016 – 0.077]
$\overline{\sigma^2_\epsilon}$	0.067[0.043 – 0.100]	0.028[0.011 – 0.058]	0.028[0.011 – 0.058]	0.028[0.011 – 0.057]	0.027[0.011 – 0.055]

($\Delta\tilde{\pi}$) will also cause deviations from true values, but these errors are ‘classical’ and will result in attenuated estimates of β. To see this, note:

$$E[\hat{\beta}] = \frac{Cov(\beta\pi_{im} + \delta_{im}, \hat{\pi}_{im})}{Var(\hat{\pi}_{im})} \quad [8]$$

Given our model, $\hat{\pi}_{im}$ has the form:

$$\begin{aligned} \hat{\pi}_{im} =& \sum_j^{n_i^{(p)}} \sum_k^{n_{ij}^{(e)}} w_{ijk}\tilde{\pi}_{ijk} \\ =& \sum_j^{n_i^{(p)}} \sum_k^{n_{ij}^{(e)}} w_{ijk}\left[c + (\Delta\pi)_i + (\Delta\pi)_{ij} + (\Delta\tilde{\pi})_{ijk}\right] \\ =& \overline{\pi}_i + \sum_j^{n_i^{(p)}} w_{ij}(\Delta\pi)_{ij} + \sum_j^{n_i^{(p)}} \sum_k^{n_{ij}^{(e)}} w_{ijk}(\Delta\tilde{\pi})_{ijk} \end{aligned} \quad [9]$$

since $c + (\Delta\pi)_i = \overline{\pi}_i$ is constant and $w_{ij}(\Delta\pi)_{ij}$ is constant over estimates from the same population, where $w_{ij} = \sum_k^{n_{ij}^{(e)}} w_{ijk}$ is the population weight. In the case where population m had at least one genetic diversity estimate, we only used these estimates to obtain $\hat{\pi}_{im}$. Then, $n_i^{(p)} = 1$ and $w_{im} = 1$ such that:

$$\hat{\pi}_{im} = \pi_{im} + \sum_k^{n_{im}^{(e)}} w_{imk}(\Delta\tilde{\pi})_{imk} \quad [10]$$

since $\pi_{im} = \overline{\pi}_i + (\Delta\pi)_{im}$. The expected regression coefficient is then:

$$E[\hat{\beta}] = \beta E\left[\frac{\sigma^2_s + \sigma^2_p}{\sigma^2_s + \sigma^2_p + \sigma^2_\epsilon}\right] \quad [11]$$

where σ^2_ϵ is the variance of $\sum_j^{n_i^{(p)}} \sum_k^{n_{ij}^{(e)}} w_{ijk}(\Delta\tilde{\pi})_{ijk}$:

$$\sigma^2_\epsilon = \sum_j^{n_i^{(p)}} \sum_k^{n_{ij}^{(e)}} w^2_{ijk}\sigma^2_{e_{ijk}} \quad [12]$$

Since we allow σ^2_e to vary across estimates in some models, σ^2_ϵ is a random variable rather than a constant and the expectation on the right-hand side of Equation 11 is taken over the distribution of σ^2_ϵ. We will refer to the expectation in Equation 11 as $R^2_{\pi_+,\hat{\pi}}$ since it is the expected squared correlation between π and the estimate of π using data from the same population.

In most cases population m does not have a genetic diversity estimate and $\hat{\pi}_{im}$ is obtained using estimates from other populations. Then,

$$E[\hat{\beta}] = \beta E\left[\frac{\sigma^2_s}{\sigma^2_s + K\sigma^2_p + \sigma^2_\epsilon}\right] \quad [13]$$

where $K_i = \sum_j^{n_i^{(p)}} w_{ij}^2$ and it is assumed that the species has a sufficiently large number of populations compared to those sampled that the covariance between the true value and the estimate is only due to the deviations of the species means. Note that if the weights are equal across populations then $K_i = 1/n_i^{(p)}$ and $K\sigma^2_p$ is the sampling variance on $\overline{\pi}_i$ from sampling a finite $(n_i^{(p)})$ number of populations. As the number of populations becomes large, K tends to zero and the attenuation only comes from estimation error - using the species-mean diversity $(\overline{\pi})$ in place of population m's diversity does not cause attenuation. The expectation on the right-hand side of Equation 13 is an expectation over both σ^2_e and K and we will refer to it as $R^2_{\overline{\pi},\hat{\pi}}$ since it is the average squared correlation between the species mean π $(\overline{\pi})$ and the estimate of π $(\hat{\pi})$ using all available data from that species.

We can also define the expected squared correlation between π in a population and our estimate of π using all available data from that species from populations other than the focal population:

$$R^2_{\pi_-,\hat{\pi}} = E\left[\frac{\sigma_s^4}{(\sigma_s^2+\sigma_p^2)(\sigma_s^2+K\sigma_p^2+\sigma_\epsilon^2)}\right] \quad [14]$$

For completeness, we can also define the expected squared correlation between pairs of estimates taken on different populations of the same species

$$R^2_{\tilde{\pi}_-,\tilde{\pi}} = E\left[\left(\frac{\sigma_s^2}{\sigma_s^2+\sigma_p^2+\sigma_e^2}\right)^2\right] \quad [15]$$

where it is assumed the error variance of the estimates are uncorrelated within species (note the expression involves the error variances of $\tilde{\pi}$ (σ^2_e) not $\hat{\pi}$ (σ^2_ϵ)).

From the main analyses, we also quantified the association between I_A and π using an R^2 value. Although the deviation of a population's diversity from the species mean does not result in attenuation of β, Berkson errors do result in a lower R^2. The true R^2 is

$$R^2_{I_A,\pi} = \frac{\beta^2 Var(\pi)}{\beta^2 Var(\pi)+Var(\delta)} \quad [16]$$

However, the expected adjusted-R^2 between I_A and $\hat{\pi}$ is

$$\begin{aligned} R^2_{I_A,\hat{\pi}} &= \tfrac{E[\hat{\beta}]^2 Var(\hat{\pi})}{\beta^2 Var(\pi)+Var(\delta)} \\ R^2_{I_A,\hat{\pi}}/R^2_{I_A,\pi} &= \tfrac{Var(\hat{\pi})}{Var(\pi)}\left(\tfrac{E[\hat{\beta}]}{\beta}\right)^2 \end{aligned} \quad [17]$$

where the use of an adjusted R^2 deals with the upward bias $E[\hat{\beta}^2] > \beta^2$ that arises from estimation error on β even when the estimator is unbiased $(E[\hat{\beta}] = \beta)$. When $\hat{\pi}$ is obtained using estimates from the focal population then,

$$\begin{aligned} R^2_{I_A,\hat{\pi}}/R^2_{I_A,\pi} &= \tfrac{\sigma_s^2+\sigma_p^2+\sigma_\epsilon^2}{\sigma_s^2+\sigma_p^2}\left(\tfrac{\sigma_s^2+\sigma_p^2}{\sigma_s^2+\sigma_p^2+\sigma_\epsilon^2}\right)^2 \\ R^2_{I_A,\hat{\pi}}/R^2_{I_A,\pi} &= R^2_{\pi_+,\hat{\pi}} \\ R^2_{I_A,\hat{\pi}} &= R^2_{I_A,\pi}R^2_{\pi_+,\hat{\pi}} \end{aligned} \quad [18]$$

When $\hat{\pi}$ is obtained using estimates from other populations then,

$$\begin{aligned} R^2_{I_A,\hat{\pi}}/R^2_{I_A,\pi} &= \tfrac{\sigma_s^2+K\sigma_p^2+\sigma_\epsilon^2}{\sigma_s^2+\sigma_p^2}\left(\tfrac{\sigma_s^2}{\sigma_s^2+K\sigma_p^2+\sigma_\epsilon^2}\right)^2 \\ R^2_{I_A,\hat{\pi}} &= R^2_{I_A,\pi}R^2_{\pi_-,\hat{\pi}} \end{aligned} \quad [19]$$

The above derivations have assumed a linear relationship between I_A and π, yet in the main analyses we assume a linear relationship between $ln(I_A)$ and $ln(\pi)$. However, under a Delta approximation

$$R^2_{ln(x),ln(y)} = \frac{Cov(ln(x),ln(y))^2}{Var(ln(x))Var(ln(y))} \approx \frac{Cov(x/\bar{x},y/\bar{y})^2}{Var(x/\bar{x})Var(y/\bar{y})} \approx R^2_{x,y} \quad [20]$$

and so the degree of attenuation can be approximated using the results of our diversity models. The variance in $\hat{\pi}$, and it's decomposition, is considerably easier to derive than the variance in $ln(\hat{\pi})$ since we average $\tilde{\pi}$ to obtain $\hat{\pi}$ and then log (i.e. we use $ln(\hat{\pi})$ rather than $\overline{ln(\tilde{\pi})}$). When estimation errors on π are symmetrical around zero then $ln(\hat{\pi})$ is a less downwardly biased estimate of $ln(\pi)$ than $\overline{ln(\tilde{\pi})}$ because the error variance on $\hat{\pi}$ is less than on $\tilde{\pi}$ when there is more than one estimate to average over.

Estimated R^2 values that do, and do not, control for systematic differences between estimates are summarised in Tables S3 and S4, respectively. While we used all available diversity estimates $(\tilde{\pi})$ to estimate the parameters of our diversity model (see Table S1) the R^2 statistics (and the average value of σ^2_ϵ) are calculated by averaging over the 108 species for which we had

Table S3. Summary of diversity R^2 values derived from models that did not control for systematic differences in π estimates. For the main analysis in the paper, a weighted average ($\hat{\pi}$) of the publication/population-level diversity estimates ($\tilde{\pi}$) are used. $R^2_{\pi,\hat{\pi}}$ measures how well our $\hat{\pi}$ estimates predict species-mean diversity. $R^2_{\pi_+,\hat{\pi}}$ and $R^2_{\pi_-,\hat{\pi}}$, measures how well our $\hat{\pi}$ estimates predict population-level diversity had all publication/population-level estimates come from the focal population, or different population(s), respectively. $R^2_{\tilde{\pi}_-,\tilde{\pi}}$ measures how well publication/population-level estimates from different populations predict each other. All R^2 values were calculated using the properties of the π estimates (pooled, genome-wide, $n^{(l)}$) used in the main analyses. Each R^2 value was calculated for each of the diversity analyses detailed in Table S1.

	Model A	Model B	Model C	Model D	Model E
$R^2_{\pi,\hat{\pi}}$	0.903[0.876 – 0.925]	0.913[0.887 – 0.935]	0.914[0.889 – 0.937]	0.915[0.888 – 0.937]	0.916[0.892 – 0.938]
$R^2_{\pi_+,\hat{\pi}}$	0.943[0.913 – 0.967]	0.955[0.925 – 0.976]	0.956[0.931 – 0.976]	0.970[0.945 – 0.987]	0.968[0.946 – 0.985]
$R^2_{\pi_-,\hat{\pi}}$	0.852[0.801 – 0.900]	0.859[0.808 – 0.909]	0.859[0.807 – 0.907]	0.844[0.791 – 0.893]	0.850[0.801 – 0.895]
$R^2_{\tilde{\pi}_-,\tilde{\pi}}$	0.752[0.689 – 0.803]	0.774[0.715 – 0.828]	0.776[0.718 – 0.833]	0.777[0.715 – 0.830]	0.780[0.723 – 0.834]

Table S4. Summary of diversity R^2 values derived from models that controlled for systematic differences between 'pooled', 'non-pooled k-mer' and 'non-pooled standard' estimates of π. For the main analysis in the paper, a weighted average ($\hat{\pi}$) of the publication/population-level diversity estimates ($\tilde{\pi}$) are used. $R^2_{\pi,\hat{\pi}}$ measures how well our $\hat{\pi}$ estimates predict species-mean diversity. $R^2_{\pi_+,\hat{\pi}}$ and $R^2_{\pi_-,\hat{\pi}}$, measures how well our $\hat{\pi}$ estimates predict population-level diversity had all publication/population-level estimates come from the focal population, or different population(s), respectively. $R^2_{\tilde{\pi}_-,\tilde{\pi}}$ measures how well publication/population-level estimates from different populations predict each other. All R^2 values were calculated using the properties of the π estimates (pooled, genome-wide, $n^{(l)}$) used in the main analyses. Each R^2 value was calculated for each of the diversity analyses detailed in Table S2.

	Model A	Model B	Model C	Model D	Model E
$R^2_{\pi,\hat{\pi}}$	0.898[0.867 – 0.919]	0.912[0.887 – 0.934]	0.913[0.888 – 0.937]	0.911[0.883 – 0.936]	0.912[0.886 – 0.937]
$R^2_{\pi_+,\hat{\pi}}$	0.943[0.913 – 0.966]	0.977[0.951 – 0.992]	0.977[0.950 – 0.990]	0.978[0.953 – 0.992]	0.978[0.955 – 0.992]
$R^2_{\pi_-,\hat{\pi}}$	0.838[0.786 – 0.889]	0.832[0.774 – 0.881]	0.832[0.772 – 0.885]	0.827[0.772 – 0.878]	0.829[0.777 – 0.883]
$R^2_{\tilde{\pi}_-,\tilde{\pi}}$	0.739[0.671 – 0.790]	0.773[0.714 – 0.825]	0.773[0.711 – 0.828]	0.769[0.707 – 0.827]	0.773[0.713 – 0.833]

both I_A observations and estimates of π. Using related models to our diversity analyses, (5) estimated $R^2_{\pi_+,\hat{\pi}}$ to be 0.789 and $R^2_{\pi_-,\hat{\pi}}$ to be 0.784 on a subset of our data.

In addition to systematic differences between k-mer based estimates and estimates made using traditional approaches, we were concerned that the estimates may not generally be comparable because they are assessing different types of polymorphism. Of the 47 species for which we had ToL estimates, 15 had an estimate using a more traditional approach (Figure S1). A simple linear regression of non-ToL estimates ($\tilde{\pi}$) on ToL estimates indicated a reasonable relationship (estimate followed by 95% confidence intervals: intercept = -0.234[-2.911 – 2.443]$\times10^{-3}$; slope = 0.660[0.488 – 0.833]; R^2 = 0.576). However, the R^2 is somewhat less than we would predict if k-mer and traditional approaches were equivalent after correcting for systematic bias ($R^2_{\tilde{\pi}_-,\tilde{\pi}}$=0.739[0.671 – 0.790], Model a). Whether the lower R^2 is due to chance, given the low number of species, or indicates k-mer based estimates assess different types of polymorphism remains unclear.

2. Empirical analyses

Univariate models. As outlined in the main text, all statistical analyses involved Bayesian linear mixed models fitted using the package MCMCglmm v2.35 (19) in R v4.4.2 (20). Scaled (by 1,000) $F_{1,1}$ priors were used for all random-effect variance components and an inverse gamma prior, with shape and scale equal to 0.002, was used for the residual variance component. Normal priors with zero mean and large variances (10^8) were used for the fixed effects. The MCMC chains were run for 500,000 iterations with a burn-in period of 100,000, sampling every 200 iterations. Significance was assessed using pMCMC values (21) or a Wald test for omnibus tests of multi-category factors where the posterior means and covariance matrix of the effects were used. Posterior distributions are summarised by their median (22) and 95% credible intervals. A threshold of 0.005 was used to determine significance and values between 0.005-0.05 were considered suggestive (23).

Molecular genetic diversity vs. Evolvability. The relationship between a population's additive genetic variance, V_A, and genetic diversity is expected to be proportional under some evolutionary scenarios (see section 3). Estimates of evolvability, $\widehat{I_A}$, and nucleotide diversity, $\hat{\pi}$, were therefore log-transformed prior to analysis. Whilst true evolvabilities cannot be negative, measurement error can produce estimates below zero. To retain these data, we first tried left-censored models, treating non-positive values as censored between $-\infty$ and -15.903 on the log-scale, where -15.903 is the log of the minimum positive value reported. However, these models fitted the data poorly. We also tried a Gaussian model with log-link using the package 'rstan' v2.32.7 (24) to accommodate negative estimates on the data scale whilst allowing a multiplicative underlying model. However, issues with chain convergence prevented useful estimation of effects and variance components. Consequently, we finally chose to simply remove non-positive estimates for the final model. Given that only a small portion of evolvability

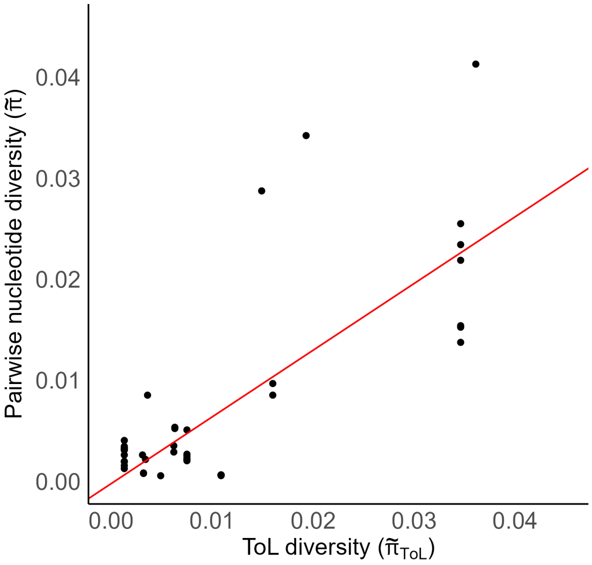

Fig. S1. k-mer diversity estimates of π ($\tilde{\pi}_{ToL}$) from the Tree of Life (ToL) data portal against published estimates of pairwise nucleotide diversity ($\tilde{\pi}$) based on multiple individuals, across 46 species. The regression line (slope = 0.66) is shown in red.

estimates were non-positive (4.54%), the exclusion of these estimates should only have a minor quantitative effect on the results of the analyses and is not expected to impact our qualitative conclusions.

True variation in $ln(I_A)$ among 108 species, was estimated by fitting $ln(\hat{\pi})$ as a covariate with regression coefficient β, and *phylogenetic* and *non-phylogenetic* species effects as random. The phylogenetic covariance structure was assumed to be proportional to the amount of time two species have shared ancestry, based on a phylogeny constructed from published divergence-time estimates (Figure 1; 25). The tree was scaled to unit length such that the estimated variance in the phylogenetic ($V_{ln(I_A)\text{:Phy}}$) and non-phylogenetic ($V_{ln(I_A)\text{:S}}$) species effects sum to give the total between-species variation in $ln(I_A)$, after accounting for $ln(\hat{\pi})$. Differences in $ln(\widehat{I_A})$ due to estimation methodology were accounted for with fixed effects for *method* and the number of *fixed* and *random effect terms*. We did not incorporate a formal measurement error structure as this would require the exclusion of all evolvability estimates lacking a reported or calculable measure of precision, limiting the precision with which model effects can be estimated. Trait differences in $ln(\widehat{I_A})$ were modelled with fixed effects for *trait category* and *trait dimension* and a random effect for *trait* identity. A random *publication* effect was fitted to capture remaining differences among studies not explained by these predictors. True among-population variance was not estimated because few species had estimates from multiple populations across multiple publications, providing little power to partition variance at these levels.

The estimated parameters and variance components are reported in Tables S5 and S6. The slope of the regression was β = 0.160 [-0.125 – 0.452] (P=0.282), therefore doubling $\hat{\pi}$ would only increase mean evolvability by 11.7% [-9.1 – 35.9]. Despite substantial variation in $ln(I_A)$ between-species (standard deviation (SD) of 1.563 [0.811 – 2.510]), only 1.1% [-2.1 – 8.7] of this could be predicted by $ln(\hat{\pi})$ (note we carry the sign in the R^2 value). The best estimates suggest that phylogenetic relatedness explained the largest proportion of among-species variation in $ln(I_A)$ (92.8% [51.6 – 100.0]) whereas residual non-phylogenetic species differences accounted for only a small proportion (4.5% [0.0 – 45.4]), however credible intervals were wide. $ln(I_A)$ did not significantly depend on the types of relatives ($\chi^2_6 = 6.367$, P=0.383) or the number of fixed effects (0.010 [-0.103 – 0.127], P=0.852), but suggestively increases with the number of random effects (0.321 [0.006 – 0.640], P=0.042). There were suggestive (23) and significant differences in $ln(I_A)$ among different trait categories ($\chi^2_4 = 11.94$, P=0.018) and dimensions ($\chi^2_5 = 28.291$, P<0.001) respectively, and there was substantial among-trait variation that was not captured by these broad classifications (SD = 1.054 [0.875 – 1.222]). There was still considerable among-publication variance after accounting for differences in estimation and trait choice (SD = 1.167 [0.941 – 1.415]), but it is unclear to what extent this represents remaining methodological differences versus true differences between populations since among-population variance was not separately estimated. There was substantial residual variation (SD = 1.365 [1.295 – 1.438]) and the residuals were slightly more leptokurtic than normal. We next repeated the analysis, substituting $ln(\hat{\pi})$ for $ln(\hat{\pi}_N/\hat{\pi}_S)$. The slope of the regression was β = 0.014 [-0.715 – 0.685] (P=0.974), therefore doubling $\hat{\pi}_N/\hat{\pi}_S$ only corresponds to an increase in evolvability by 1.0% [-39.1 – 60.8] and 0.0% [-9.9 – 20.2] of true variation in $ln(I_A)$ between species (n = 37) could be predicted by $ln(\hat{\pi}_N/\hat{\pi}_S)$.

Microsatellite diversity has historically been used to infer the amount of neutral genetic variation within species (26). In a prior study (5), only a small number of species had both microsatellite H_e and I_A estimates, which prevented precise inference of the relationship between these measures. Here, we find no clear association between $ln(I_A)$ and $ln(\widehat{H_e})$ across the 65 species with estimates of both measures. The slope of the regression was β = -0.473 [-1.334 – 0.258] (P=0.239), therefore doubling $\widehat{H_e}$ may reduce mean evolvability, 2^β = -28.0% [-62.0 – 16.7], but credible intervals are wide. The R^2 was effectively zero (-0.8% [-10.4 – 1.1]). A parallel analysis restricted to 40 species where both H_e and I_A were estimated in the same population

produced similarly uncertain results. The slope of the regression was β = -0.019 [-1.186 – 1.171] (P=0.973), suggesting that evolvability is only expected to decrease marginally if $\widehat{H_e}$ doubled (2^β = -1.3% [-65.4 – 108.2]) and the R^2 was again effectively zero (-0.0% [-16.2 – 20.9]).

Molecular genetic diversity vs. Heritability. Given the weak correlation between I_A and h^2 (Fig. S2; 2), we performed additional analyses to quantify the relationship between the three measures of molecular genetic variation and h^2. Although quantitative genetic theory predicts a log-log linear relationship between V_A and genetic diversity, it is not clear whether this expectation holds when V_A is variance-standardised (i.e., when expressed as heritability, h^2). h^2 has a low dynamic range because it is typically bounded between 0-1, meaning that the results are unlikely to differ appreciably with- or without log-transformation of h^2. Therefore, we did not log-transform heritability and non-positive values were retained (7.4% of estimates). The priors, MCMC specifications and model structure are otherwise identical to the univariate models described above. As with the evolvability models, the relationships between h^2 and each of $ln(\hat{\pi})$, $ln(\hat{\pi}_N/\hat{\pi}_S)$ and $ln(\widehat{H_e})$ were assessed individually. Across all heritability models, we only report the R^2 since a log-log regression would be required to quantify the expected proportional change in h^2 if genetic diversity were doubled (2^β).

The parameters and variance components estimated in the univariate analysis of h^2 and $ln(\hat{\pi})$ are reported in Tables S7 and S8. The regression slope was β =-0.023 [-0.055 – 0.011] (P=0.193), and despite moderate between-species ($n = 130$) variation in h^2 (standard deviation (SD) of 0.136 [0.077 – 0.286]), very little -3.2% [-21.8 – 2.1] of this could be predicted by $ln(\hat{\pi})$. There was low power to partition the total among-species variation in h^2 between phylogenetic (44.8% [0.0 – 97.7]) and residual non-phylogenetic species components (48.8% [0.0 – 92.5]). Substituting $ln(\hat{\pi})$ for $ln(\hat{\pi}_N/\hat{\pi}_S)$ yielded similar conclusions, with β =0.031 [-0.037 – 0.094] (P=0.360) and R^2 = 2.1% [-4.3 – 37.9] ($n = 49$ species).

The relationship between h^2 and microsatellite $ln(\widehat{H_e})$ was significantly positive, with β =0.088 [0.066 – 0.110](P<0.5×10^{-3}) and R^2 = 4.3% [0.4 – 9.1]. The parallel analysis restricted to species where both H_e and h^2 were estimated in the same population produced very similar results, with β =0.091 [0.067 – 0.114] (P<0.5×10^{-3}) and R^2 = 2.5% [0.1 – 6.5]. However, consistent with the findings of (5), this association was largely driven by a single study on *Arabidopsis thaliana* (27). When data from this study were excluded (140 h^2 estimates from 12 populations), the estimated relationship was weaker with wider credible intervals, both when heterozygosity estimates from different populations were included (β =-0.030 [-0.124 – 0.061], P=0.534 and R^2 = -0.5% [-9.5 – 4.9]) and excluded (β =-0.014 [-0.157 – 0.137], P=0.848 and R^2 = -0.1% [-9.7 – 8.6]).

Quantitative genetic variation vs. IUCN Red List status. In recent years, there has been renewed discussion of whether genetic data should inform assessments of species' Red List status and prioritisation of conservation efforts (28, 29), and whilst studies have reported (albeit weak) associations between Red List category and molecular measures of genetic diversity (28, 30), no such analyses have been reported for levels of additive genetic variance.

Species' Red List status was recorded from the IUCN website (31). Univariate models of $ln(I_A)$ and h^2 were refitted with Red List category (least concern, near threatened, vulnerable, endangered or critically endangered) as a predictor, excluding molecular genetic diversity. The sample size per category is given in Table S9. We did not find significant differences in mean I_A or h^2 across IUCN Red List categories. (I_A: χ^2_4 = 0.264, P=0.992, Table S10; h^2: χ^2_4 = 6.279, P=0.179, Table S11). Given the paucity of estimates for species of conservation concern according to the Red List, both models were repeated with a condensed classification of extinction risk in which all at-risk categories were grouped into a single 'conservation concern' group and compared to species of 'least concern'. Mean I_A was 3.5% [-54.8 – 84.8] (P=0.911) higher and mean h^2 was 0.08 [-0.01 – 0.16] (P=0.053) higher in species of conservation concern. However, given the credible intervals on these differences remain wide, we consider this an open question.

Bivariate models. In many species with estimates of quantitive genetic variation, estimates of molecular genetic diversity were not available. In order to leverage information from these species, we performed equivalent bivariate analyses for each of the molecular vs. quantitative genetic univariate models described above. In these models, both the trait-level quantitative genetic variation estimates ($ln(\widehat{I_A})$ or $\widehat{h}^2$) and species-level molecular genetic diversity estimates ($ln(\hat{\pi})$, $ln(\hat{\pi}_N/\hat{\pi}_S)$ or $ln(\widehat{H_e})$) were included as response variables. The model for the quantitative genetic response variable was equivalent to that of the respective univariate analysis but without molecular genetic diversity fitted as a predictor. For the molecular diversity response, variance among species was partitioned into a phylogenetic ($V_{M:Phy}$) and a non-phylogenetic 'residual' species ($V_{M:S}$) component. Correspondingly, the covariances between the two responses was estimated for the phylogenetic effects (Cov_{Phy}) and for the non-phylogenetic effects (Cov_S). Note that Cov_S is the covariance between the species effects for quantitative genetic variation estimates and the residuals for molecular genetic variation estimates (since we only use one estimate per species) and so a 'covu' approach was used (32). The overall species-level regression coefficient was obtained as

$$\beta = \frac{Cov_S + Cov_{Phy}}{V_{M:S} + V_{M:Phy}} \qquad [21]$$

Unlike the univariate analyses described above and in the main text, which implicitly assumes that the regression of quantitative genetic variation on molecular genetic diversity is the same at both phylogenetic and non-phylogenetic levels, the bivariate model allows the regression to be different. In the presence of phylogenetic effects, the bivariate model is expected to leverage additional information from species that only have estimates of quantitative genetic variation but for which estimates of molecular diversity exist for closely related taxa. While this may be expected to increase the precision with which the relationship between quantitative and molecular genetic variation can be estimated, this may be offset by allowing the regression

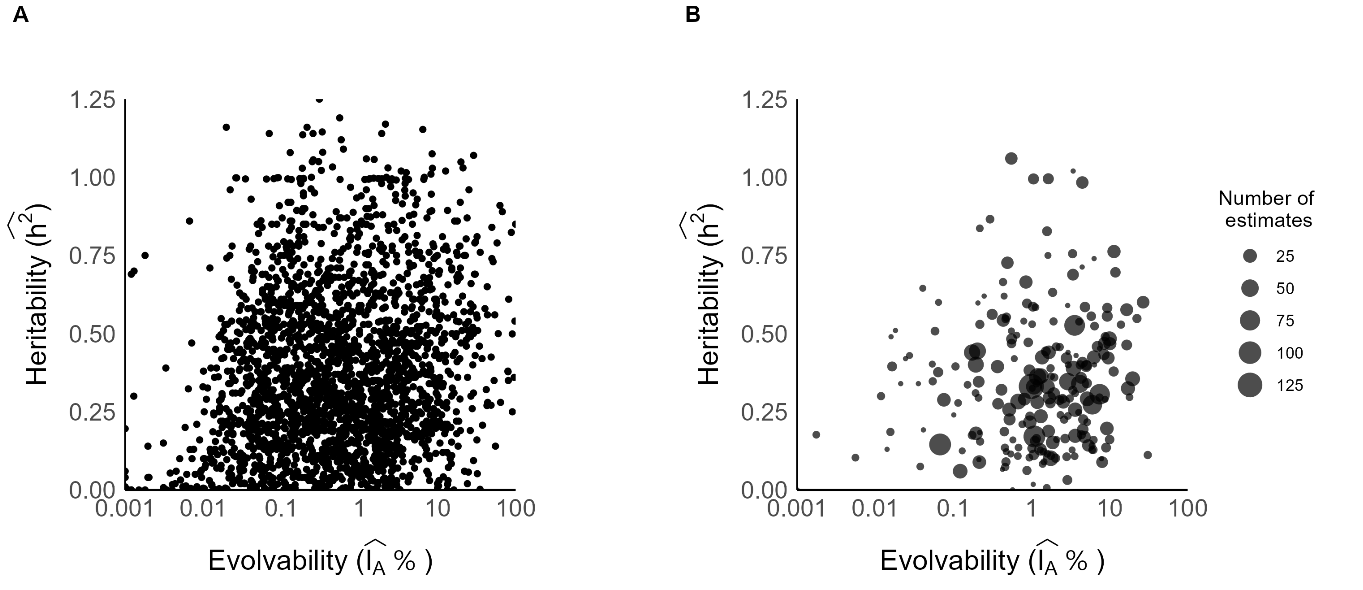

Fig. S2. Measured heritability, $\widehat{h^2}$, against evolvability, $\widehat{I_A}$, for **(A)** 1,976 traits and **(B)** averaged across traits for 183 species, where the size of the point shows the number of estimates over which the average was calculated.

at the two levels to differ rather than pooling information across these levels. Indeed, the credible intervals on the regression slope are wider than in the univariate analysis, and this appears to be due to the large uncertainty on the phylogenetic (co)variances. Parameters relating solely to quantitative genetic variation are, however, expected to always be more precise since more data are used. Priors and MCMC specifications are the same as for the univariate models except the prior for the species covariance matrix was an inverse-Wishart distribution that induced marginal inverse-gamma priors on the variances with shape and scale equal to 0.002.

Molecular genetic diversity vs. Evolvability. The parameters estimated in the bivariate analysis of $ln(I_A)$ and $ln(\hat{\pi})$ (Tables S12 and S13) were broadly consistent with those of the univariate model. The association between $ln(I_A)$ and $ln(\hat{\pi})$ was again found to be weak (β =-0.047 [-0.629 – 0.586], P=0.877; 2^{β} = -3.2% [-41.1 – 41.6] and R^2 =-0.2% [-20.6 – 14.6]). The between-species (n = 193) variation in $ln(I_A)$ (standard deviation (SD) of 1.724 [1.049 – 2.683]) was mostly comprised of phylogenetic species effects (85.0% [62.1 – 97.2]), and residual non-phylogenetic species effects only accounted for a small proportion (9.0% [1.1 – 32.5]) of true variation among-species. $ln(I_A)$ did not significantly differ among *types of relatives* (χ^2_6 = 11.658, P=0.070), the number of *fixed effects* (0.010 [-0.078 – 0.098], P=0.809), or the number of *random effects* (0.159 [-0.065 – 0.385], P=0.162). $ln(I_A)$ significantly differed among different *trait categories* (χ^2_4 = 23.073, P<0.001) and *dimensions* (χ^2_5 = 45.866, P<0.001), and there was substantial among-trait variation that was not captured by these broad classifications (SD = 0.985 [0.841 – 1.120]). There was considerable among-publication variance after accounting for differences in estimation and trait choice (SD = 1.055 [0.877 – 1.266]) and substantial residual variation (SD = 1.442 [1.385 – 1.502]). The association between $ln(I_A)$ and $ln(\hat{\pi})$ remained weak when the bivariate analysis was repeated with 'pooled', 'non-pooled k-mer' and 'non-pooled standard' effects fitted as fixed to account for systematic differences in $ln(\hat{\pi})$ (see section 1). $ln(\hat{\pi})$ was not significantly different when estimated from samples of pooled populations as opposed to unpooled (0.332 [-0.132 – 0.791], P=0.143), but was significantly larger when estimated using k-mer based approaches (0.948 [0.483 – 1.457], P<0.5×10^{-3}). Nevertheless, the results were comparable to those of our standard bivariate model in which systematic differences in $ln(\hat{\pi})$ were not accounted for, with the slope estimated to be β =-0.130 [-0.819 – 0.471], P=0.694, 2^{β} = -8.6% [-46.3 – 34.5] and R^2 =-0.9% [-23.6 – 9.4].

The association between $ln(I_A)$ and $ln(\hat{\pi}_N/\hat{\pi}_S)$ appeared stronger than in the univariate analysis, but remained too uncertain to be useful (β =0.642 [-0.162 – 1.502], P=0.140; 2^{β} = 56.1% [-22.7 – 154.9] and R^2 =14.3% [-2.2 – 37.0]). The relationship between $ln(I_A)$ and microsatellite $ln(\widehat{H_e})$ was again weak with wide credible intervals, both when diversity estimates from different populations were included (β =-0.965 [-2.843 – 0.536], P=0.183; 2^{β} = -48.8% [-91.1 – 25.5] and R^2 = -5.8% [-30.2 – 2.8]), and excluded from the analysis (β = -0.369 [-2.438 – 1.460], P=0.632; 2^{β} = -22.5% [-94.5 – 114.6] and R^2 = -0.7% [-24.0 – 11.1]).

Molecular genetic diversity vs. Heritability. The parameters and variance components estimated in the bivariate analysis of h^2 and $ln(\hat{\pi})$ are reported in Tables S14 and S15. The regression slope was β =-0.009 [-0.039 – 0.015] (P=0.415), and little of the between-species (n = 246) variation in h^2 (standard deviation (SD) of 0.162 [0.136 – 0.192]) could be predicted by $ln(\hat{\pi})$ (R^2 = -0.7% [-9.1 – 1.7]). Best estimates suggest that phylogenetic relatedness explained a small proportion of between-species

variation in h^2 (3.0% [0.0 – 24.6]) and that residual between-species differences contributed substantially (95.3% [70.9 – 100.0]).

There was suggestive evidence that h^2 differs among types of relatives (χ^2_6 = 15.56, P=0.016), and decreases with an increasing number of fixed (-0.013 [-0.023 – -0.004], P=0.010) and random effects (-0.025 [-0.048 – -0.004], P=0.024). h^2 significantly differed among different trait categories (χ^2_4 = 23.418, P<0.001) but not dimensions (χ^2_5 = 7.826, P=0.166), and there was considerable among-trait variation that was not captured by these broad classifications (SD = 0.078 [0.066 – 0.092]). There was substantial among-publication variance after accounting for differences in estimation and trait choice (SD = 0.135 [0.116 – 0.153]) and substantial residual variation (SD = 0.201 [0.196 – 0.207]). For $ln(\hat{\pi}_N/\hat{\pi}_s)$, the regression slope was again weak (β =-0.010 [-0.039 – 0.015], P=0.386) and variation in $ln(\hat{\pi}_N/\hat{\pi}_s)$ explained very little of the variation in h^2 (R^2 = -0.7% [-8.1 – 2.8]).

Likewise, models with microsatellite $ln(\widehat{H_e})$ included as the molecular diversity predictor showed no detectable association with h^2, both when diversity estimates obtained from different populations were included (β =0.003 [-0.149 – 0.158], P=0.968 and R^2 = 0.0% [-5.7 – 7.3]) and excluded from the analyis (β =0.013 [-0.107 – 0.131], P=0.829 and R^2 = 0.1% [-5.0 – 6.8]).

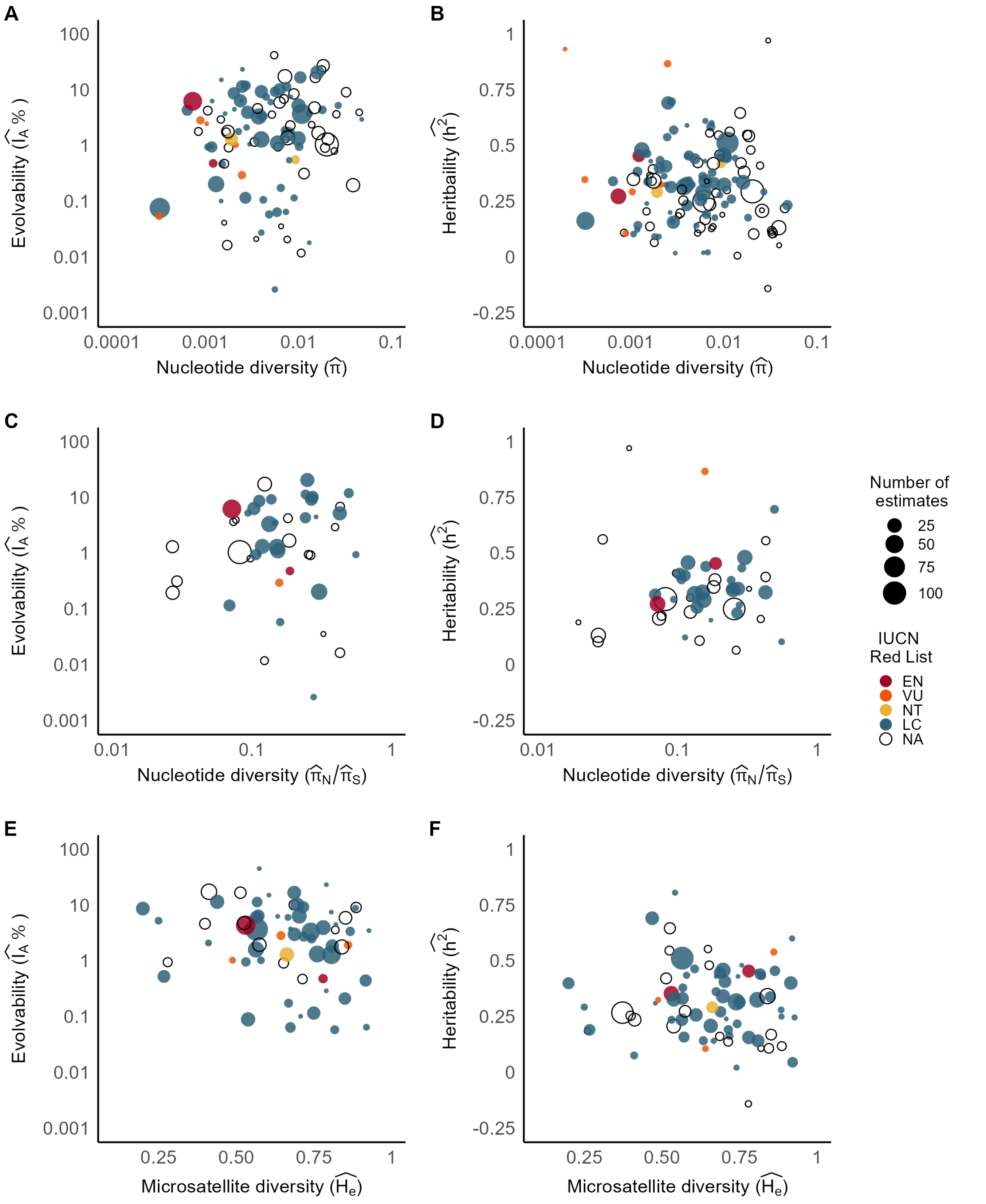

Fig. S3. Measured species-mean quantitative genetic variation against molecular genetic variation: **(A)** Evolvability, $\widehat{I_A}$, and putatively neutral nucleotide diversity, $\hat{\pi}$ (n =108); **(B)** Heritability, $\widehat{h^2}$, and putatively neutral nucleotide diversity, $\hat{\pi}$ (n =130); **(C)** Evolvability and the ratio of nonsynonymous to synonymous nucleotide diversity, $\hat{\pi}_N/\hat{\pi}_S$ (n =44); **(D)** Heritability and the ratio of nonsynonymous to synonymous nucleotide diversity (n =49); **(E)** Evolvability and microsatellite expected heterozygosity, $\widehat{H_e}$ (n =65); **(F)** Heritability and microsatellite expected heterozygosity (n =75). The size of the points shows the number of estimates over which the average evolvability is calculated and the colour shows the IUCN Red List category for that species, where EN = Endangered, VU = Vulnerable, NT = Near threatened, LC = Least concern, and NA = Not classified.

3. Theoretical expectations

In this section, we outline the expected relationship between additive genetic variance (V_A) and neutral genetic diversity (π) under models of drift-selection-mutation balance. Throughout we assume additive effects on trait values and linkage-equilibrium. The models are covered in depth in Chapters 24 and 28 of 33.

Equilibrium: Under neutrality. Under mutation-drift balance the expected V_A depends on the mutation model. Under the standard incremental model of mutation and additive effects (34),

$$V_A = 2N_eV_m \quad [22]$$

where N_e is the effective population size and V_m the mutational variance of the trait. Since the following models assume linkage-equilibrium it is typical to treat a locus as a region of length L with mutation rate $L\mu$, where μ is the per-site mutation rate. Then, $V_m = 2nL\mu E[m^2]$ where n is the number of loci contributing to the trait and $E[m^2]$ is the average squared effect of a new mutation on the trait. Under neutrality

$$\pi = 4N_e\mu/(1 + 4N_e\mu) \quad [23]$$

which is approximately $4N_e\mu$ when small, such that

$$\begin{aligned} ln(V_A) = & \; ln(4N_e\mu) + ln(LnE[m^2]) \\ = & \; ln(\pi) + ln(L) + ln(n) + ln(E[m^2]) \end{aligned} \quad [24]$$

and we expect a one-to-one relationship between $ln(V_A)$ and $ln(\pi)$ unless π strongly covaries with L, n and/or $E[m^2]$ over species/populations. Note that we also expect a one-to-one relationship between $ln(I_A)$ and $ln(\pi)$ since $I_A = V_A/\bar{x}^2$, and therefore

$$ln(I_A) = ln(\pi) + ln(L) + ln(n) + ln(E[m^2]) - 2ln(\bar{x}) \quad [25]$$

where $\bar{x}$ is the trait mean. Importantly, $2ln(\bar{x})$ serves as a constant in this equation. In the context of our statistical analyses, $\bar{x}$ clearly does vary, and for traits such as body-size it will almost certainly scale with π through N_e (and possibly μ). This may induce a relationship between $ln(V_A)$ and π that arises simply as a matter of scale and working with $ln(I_A)$ is effectively like treating $2ln(E[\bar{x}])$ as an offset in an analysis of $ln(V_A)$ that corrects for difference in scale.

Equilibrium: Under stabilising selection. At the other extreme, we have models of mutation-selection balance in populations large enough that the effects of genetic drift are negligible. Here, the introduction of new mutational variance is counteracted by the strength of stabilising selection acting on genotypic values, denoted $1/V_S$. $V_S = \omega^2 + V_E$ where ω is the standard deviation of a Gaussian fitness function and V_E is the environmental variation of the trait (typically set to one without loss of generality). Under continuum of alleles models there are two approximate solutions for the equilibrium additive genetic variance. When selection is strong relative to mutation we have the House-of-Cards approximation (35):

$$V_A = 4nL\mu V_S \quad [26]$$

a result that is in agreement with earlier biallelic models (36) with a domain of applicability of $20L\mu \leq E[m^2]/V_S$. Under pure stabilising selection, where the trait mean is at the optimum, this domain of applicability translates into $10L\mu \leq -E[s]$, where $E[s]$ is the average selection coefficient on a new mutation which is approximately $-E[m^2]/2V_S$ (37). Under this model,

$$ln(V_A) = ln(4L) + ln(\mu) + ln(n) + ln(V_S) \quad [27]$$

Here the regression of $ln(V_A)$ on $ln(\pi)$,

$$\beta = \frac{COV(ln(V_A), ln(\pi))}{VAR(ln(\pi))}, \quad [28]$$

reduces to

$$\beta = \frac{VAR(ln(\mu)) + COV(ln(\mu), ln(N_e))}{VAR(ln(\mu)) + VAR(ln(N_e)) + 2COV(ln(\mu), ln(N_e))} \quad [29]$$

again under the assumption that n and V_S are independent of N_e and μ. If all of the variation in π is driven by differences in mutation rate, then $\beta = 1$ as in the neutral case. However, when there is substantial variation in N_e, $\beta < 1$ and populations/species with higher π will not show proportionally higher V_A.

When mutation is strong relative to selection we have the Gaussian approximation (38):

$$\begin{aligned} V_A = & \; \sqrt{2nV_SV_m} \\ = & \; \sqrt{4n^2V_SL\mu E[m^2]} \end{aligned} \quad [30]$$

Under this model,

$$\begin{aligned} ln(V_A) = & \; ln(\sqrt{4n^2 V_S L \mu E[m^2]}) \\ = & \; \tfrac{1}{2} ln(4n^2 V_S L \mu E[m^2]) \\ = & \; \tfrac{1}{2} ln(4L) + ln(n) + \tfrac{1}{2} ln(\mu) + \tfrac{1}{2} ln(V_S) + \tfrac{1}{2} ln(E[m^2]) \end{aligned} \quad [31]$$

Following a similar logic as above

$$\beta = \frac{1}{2} \frac{VAR(ln(\mu)) + COV(ln(\mu), ln(N_e))}{VAR(ln(\mu)) + VAR(ln(N_e)) + 2COV(ln(\mu), ln(N_e))} \quad [32]$$

and $\beta = \frac{1}{2}$ when all of the variation in π is driven by differences in mutation rate.

In conclusion, in extremely large populations, the relationship between π and V_A is expected to be weak if variation in mutation rate is small relative to variation in N_e. However, if variation in mutation rate is very large relative to variation in N_e then the regression should lie between 1/2 (when selection is weak compared to mutation) and one (when selection is strong compared to mutation).

Models of mutation-selection-drift balance in populations that are small enough for genetic drift to have non-negligible effects produce outcomes intermediate between the neutral case and the pure mutation-selection models. However, deriving analytical results for the expected regression coefficient is more difficult because the equilibrium V_A is not log-linear in $ln(N_e)$ (or $ln(\mu)$ under the Gaussian approximation). Instead, we can derive the partial derivatives of $ln(V_A)$ with respect to $ln(\mu)$ and $ln(N_e)$ to quantify how strongly V_A responds to proportional changes in these individual components, and subsequently gain insight into the scenarios where V_A is expected to scale strongly with π. When $ln(V_A)$ is linearly related to $ln(\mu)$ and $ln(N_e)$ and/or $ln(\mu)$ and $ln(N_e)$ are multivariate normal, the expected regression coefficients for $ln(\mu)$ and $ln(N_e)$ are equal to the expected partial derivatives over the distribution of $ln(\mu)$ and $ln(N_e)$ (39). If $ln(\mu)$ and $ln(N_e)$ do not vary too much, then the actual regression coefficients will not deviate too much from these expectations, or from the partial derivatives evaluated at the mean values of $ln(\mu)$ and $ln(N_e)$. Then, the expected regression coefficient is approximately:

$$\begin{aligned} \beta \approx & \; \frac{COV\left(ln(\mu) \frac{\partial ln(V_A)}{\partial ln(\mu)}\Big|_{\overline{ln(\mu)}} + ln(N_e) \frac{\partial ln(V_A)}{\partial ln(N_e)}\Big|_{\overline{ln(N_e)}}, ln(\mu) + ln(N_e)\right)}{VAR(ln(\mu)) + VAR(ln(N_e)) + 2COV(ln(\mu), ln(N_e))} \\ & \; \frac{VAR(ln(\mu)) \frac{\partial ln(V_A)}{\partial ln(\mu)}\Big|_{\overline{ln(\mu)}} + VAR(ln(N_e)) \frac{\partial ln(V_A)}{\partial ln(N_e)}\Big|_{\overline{ln(N_e)}} + COV(ln(N_e), ln(\mu)) \left[\frac{\partial ln(V_A)}{\partial ln(\mu)}\Big|_{\overline{ln(\mu)}} + \frac{\partial ln(V_A)}{\partial ln(N_e)}\Big|_{\overline{ln(N_e)}}\right]}{VAR(ln(\pi))} \end{aligned} \quad [33]$$

where $\overline{ln(\mu)}$ and $\overline{ln(N_e)}$ are the average values of $ln(\mu)$ and $ln(N_e)$. When $ln(\mu)$ and $ln(N_e)$ are uncorrelated this reduces to

$$\beta \approx \frac{VAR(ln(\mu)) \frac{\partial ln(V_A)}{\partial ln(\mu)}\Big|_{\overline{ln(\mu)}} + VAR(ln(N_e)) \frac{\partial ln(V_A)}{\partial ln(N_e)}\Big|_{\overline{ln(N_e)}}}{VAR(ln(\pi))}. \quad [34]$$

Note that the correlation between $ln(\mu)$ and $ln(N_e)$ is most likely negative (40) in which case the actual regression will be shallower than this equation predicts since both partial derivatives are expected to be non-negative.

When selection is strong relative to mutation we have the House-of-Cards approximation (41):

$$V_A = \frac{4nL\mu V_S}{1 + V_S/(N_e E[m^2])} \quad [35]$$

therefore, the partial derivative with respect to $ln(\mu)$ is simply one

$$\frac{\partial ln(V_A)}{\partial ln(\mu)} = 1 \quad [36]$$

For $ln(N_e)$ we have

$$\frac{\partial ln(V_A)}{\partial ln(N_e)} = \frac{\frac{V_S}{E[m^2]}}{N_e + \frac{V_S}{E[m^2]}} \quad [37]$$

which is a logistic function with a value of one as N_e tends to zero, a growth rate of minus one and a midpoint (i.e. when $N_e = 1$) of $ln(V_S/E[m^2])$. Consequently, at low N_e a one-to-one relationship is expected between $ln(V_A)$ and $ln(\pi)$ since both quantities scale with μ and N_e. The range of N_e over which the relationship between $ln(V_A)$ and $ln(\pi)$ remains strong is dictated by $V_S/E[m^2]$. Specifically, when $4N_e < V_S/E[m^2]$ the partial derivative with respect to $ln(N_e)$ exceeds 0.8 (Fig. S4). Note that this inequality corresponds to the scenario where $|4N_e E[s]| < 1$ and most new mutations are effectively neutral, as expected (33).

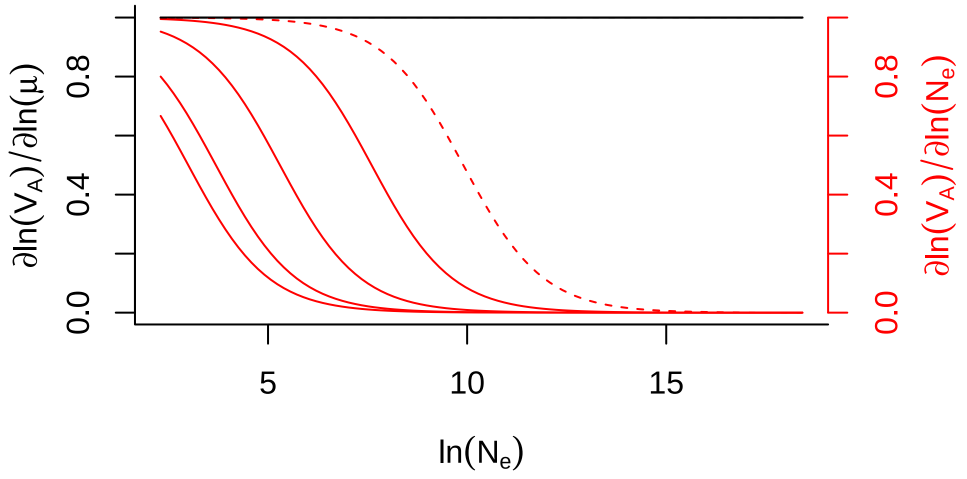

Fig. S4. Partial derivatives of $ln(V_A)$ with respect to $ln(\mu)$ (black) and $ln(N_e)$ (red) as a function of $ln(N_e)$ under the House-of-Cards approximation where selection is strong relative to mutation. The derivative with respect to $ln(N_e)$ only depends on $V_S/E[m^2]$ which we set to 5, 10, 50, 500 and 5,000 corresponding to red lines from left to right. Under pure stabilising selection, these values correspond to average selection coefficients of -0.100, -0.050, -0.010, -0.001 and -0.0001 respectively. Solid lines indicate parameter combinations where the House-of-cards approximation is expected to hold ($20L\mu \leq E[m^2]/V_S$) and dashed lines not.

When selection is relatively weak we have the Gaussian approximation (37):

$$V_A = \sqrt{\left(\frac{nV_S - 4n^2N_eL\mu E[m^2]}{2(N_e+n)}\right)^2 + \frac{4n^2N_eL\mu E[m^2]V_S}{N_e+n}} - \frac{nV_S - 4n^2N_eL\mu E[m^2]}{2(N_e+n)} \quad [38]$$

Note that we use the original derivation in (37) rather than the similar but simpler approximation in 33 (Equation 28.32c) which induces unintended behaviour in the derivatives. Nevertheless, the partial derivatives under the original derivation in 37 are ugly. With respect to $ln(\mu)$ the partial derivative is

$$\frac{\partial ln(V_A)}{\partial ln(\mu)} = 1 - \frac{V_mN_e\left(V_S - Q\right)}{SQ} \quad [39]$$

and for $ln(N_e)$ the derivative is

$$\frac{\partial ln(V_A)}{\partial ln(N_e)} = P\left(1 + \frac{N_e}{S}\left[V_m + \frac{V_S}{2n} - \frac{V_mV_S}{Q}\right]\right) \quad [40]$$

where

$$A = \frac{V_S}{2} - V_mN_e, \quad S = \sqrt{A^2 + 2V_mN_eV_S/P}, \quad Q = P(S + A) \text{ and } P = \frac{n}{N_e + n}. \quad [41]$$

In Figure S5 we plot these derivatives assuming $V_S = 20$ and $V_m = 0.001$ (42) but for a range of selection coefficients determined by n. When N_e and/or selection coefficients are small the relationship between $ln(V_A)$ and $ln(\mu)$ is one, as seen under the House-of-Cards approximation. Only under strong selection or very large population sizes does the relationship between $ln(V_A)$ and $ln(\mu)$ tend to a half, as in the deterministic Gaussian approximation. However, such scenarios probably lie outside the domain of applicability of the Gaussian approximation (since mutation is no longer strong relative to selection), so in practice the relationship between $ln(V_A)$ and $ln(\mu)$ is likely to equal one. As with the House-of-Cards approximation, the relationship between $ln(V_A)$ and $ln(N_e)$ is one when N_e is small and tends to zero as N_e and/or selection coefficients increase in magnitude. However, with the Gaussian approximation, V_A remains dependent on N_e over a greater range of values; for instance, the V_A of a highly polygenic trait under weak stabilising selection may show strong dependence on N_e if $N_e < 20,000$.

In conclusion, a strong one-to-one relationship between $ln(V_A)$ and $ln(\pi)$ is only expected when quantitative traits are neutral. If they are under stabilising selection, any relationship between $ln(V_A)$ and $ln(\pi)$ is likely driven by their shared dependence on the mutation rate and so the relationship is probably weak if the main contribution to variation in π is variation in N_e. However, if N_e is very small and/or the strength of selection on mutations that affect quantitative traits is very weak, then the

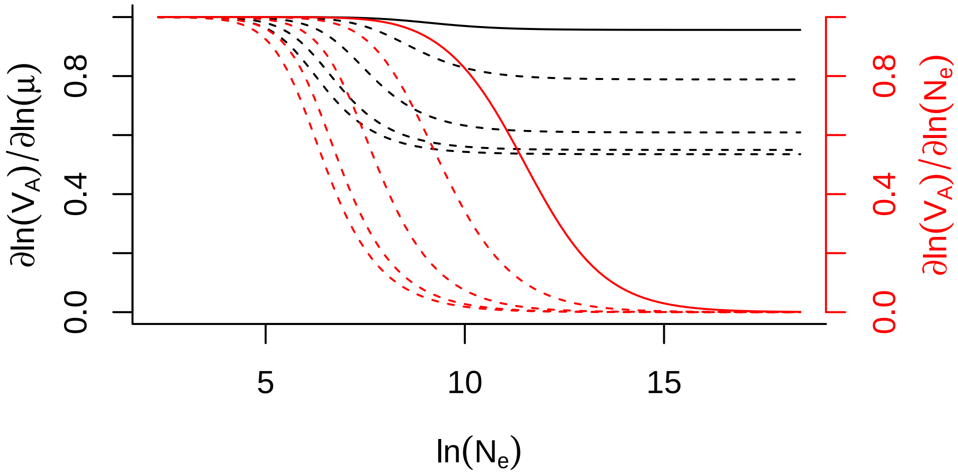

Fig. S5. Partial derivatives of $ln(V_A)$ with respect to $ln(\mu)$ (black) and $ln(N_e)$ (red) as a function of $ln(N_e)$ under the Gaussian approximation where selection is weak relative to mutation. The derivatives depend in complicated ways on V_S, V_m and n. In all cases $V_S = 20$ and $V_m = 0.001$. However, we vary n from 100, 200, 1000, 10000 and 100000 corresponding to red and black lines from left to right. Given a mutation rate of 10^{-8} and L=1,000 these values correspond to selection coefficients of -0.100, -0.050, -0.010, -0.001 and -0.0001, respectively. Solid lines indicate parameter combinations where the deterministic (i.e $N_e = \infty$) Gaussian approximation is expected to hold ($20L\mu \geq E[m^2]/V_S$) and dashed lines not. However, the domain of applicability of the Gaussian approximation is expected to be smaller when drift is higher (43).

relationship between $ln(V_A)$ and $ln(\pi)$ also has a contribution from their shared dependence on N_e and the relationship may be stronger. However, the dependence of V_A on N_e is quickly lost if N_e exceeds a few hundred, at least under the House-of-cards approximation where selection is assumed strong relative to mutation. While the Gaussian approximation predicts that this dependence remains strong for N_e of a few thousand, the House-of-cards approximation has historically been viewed to be more accurate for most systems (35). However, it is not clear whether this view remains valid in light of recent data (e.g. 44).

Non-equilibrium: Under neutrality. The above results apply to populations at equilibrium. In non-equilibrium populations, such as those that have undergone a recent bottleneck or expansion, it has been suggested that the dynamics of π and V_A are on sufficiently different time scales that the two measures may be weakly related, even in small populations (46, 47). To explore these non-equilibrium dynamics, we use recurrence equations which describe how the value of V_A or π at generation t depends on its value in the previous generation, $t-1$. The recurrence equation for V_A assuming the trait is neutral is (34)

$$V_A(t) = \quad V_A(t-1)\left(1 - \frac{1}{2N_e(t-1)}\right) + V_m \qquad [42]$$

For a more extensive treatment with dominance see (48). The recurrence equation for π is (49):

$$\begin{aligned} \pi(t) &= \quad 1 - (1-\mu)^2\left[1 - \pi(t-1)\left(1 - \frac{1}{2N_e(t-1)}\right)\right] \\ \pi(t) &= \quad (1-\mu)^2\pi(t-1)\left(1 - \frac{1}{2N_e(t-1)}\right) + \mu(2-\mu) \end{aligned} \qquad [43]$$

When μ and π are small, terms in μ^2 and $\mu\pi$ can be ignored, giving

$$\pi(t) = \quad \pi(t-1)\left(1 - \frac{1}{2N_e(t-1)}\right) + 2\mu \qquad [44]$$

If at time $t-1$, $V_A(t-1) = \pi(t-1)V_m/2\mu$, then

$$\begin{aligned} V_A(t) &= \quad \frac{V_m}{2\mu}\pi(t-1)\left(1 - \frac{1}{2N_e(t-1)}\right) + V_m \\ V_A(t) &= \quad \frac{V_m}{2\mu}\left(\pi(t-1)\left(1 - \frac{1}{2N_e(t-1)}\right) + 2\mu\right) \\ V_A(t) &= \quad \frac{V_m}{2\mu}\pi(t) \end{aligned} \qquad [45]$$

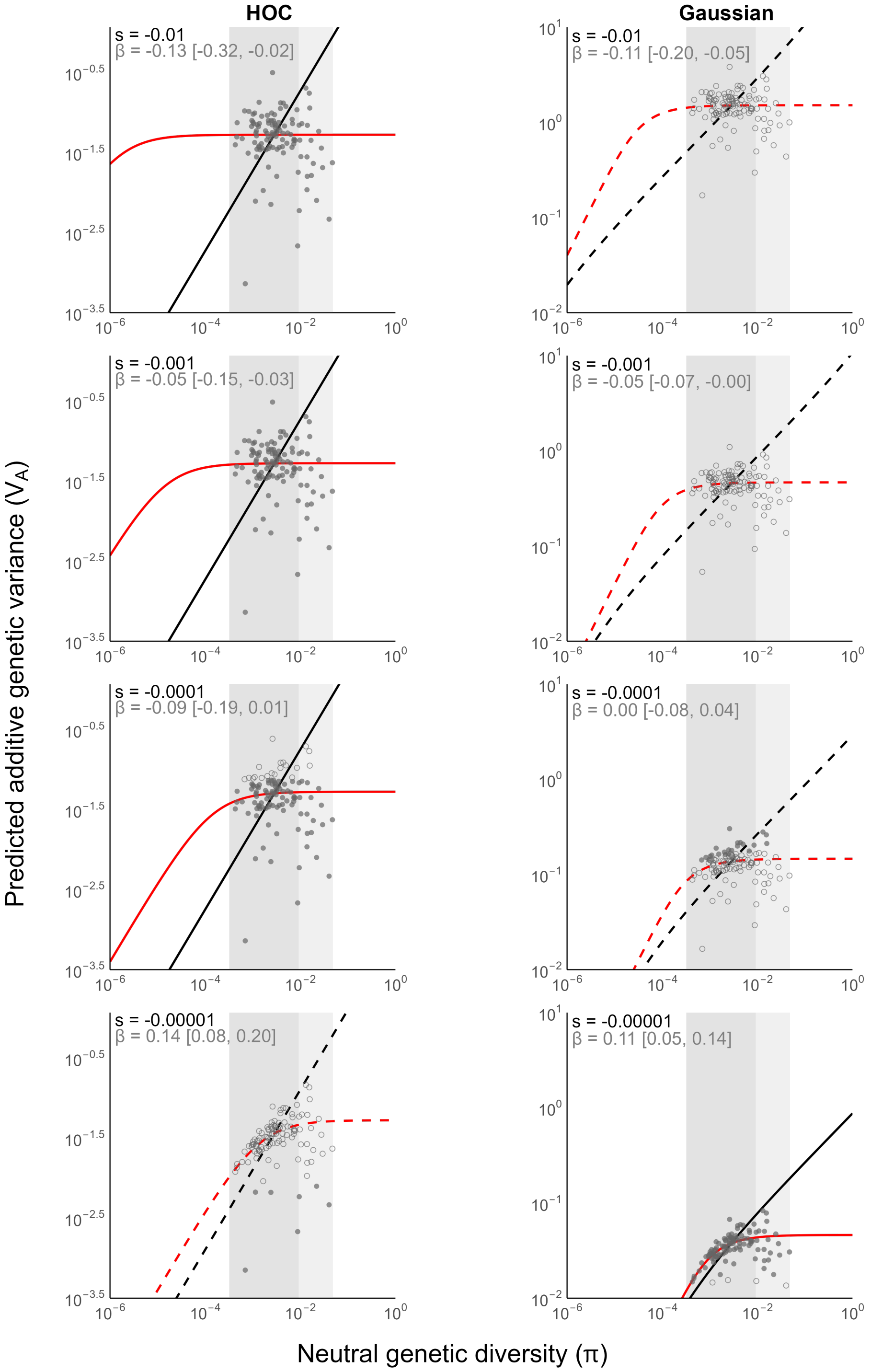

Fig. S6. The predicted relationship between neutral genetic diversity ($\pi = 4N_e\mu$) and additive genetic variance (V_A) at mutation-selection-drift equilibrium. The left column shows predictions obtained with the House-of-Cards (HOC) approximation (41) in which selection is strong relative to mutation and V_A is given by equation 35. The right column shows predictions obtained with the Gaussian approximation (37) in which selection is weak relative to mutation and V_A is given by equation 38. Rows correspond to successively weaker stabilising selection with average selection coefficients, s, of -0.01, -0.001, -0.0001 and -0.00001. Circles show the predicted equilibrium V_A for 110 multicellular eukaryotic species calculated from empirical estimates of their effective population size, N_e, and per-site mutation rate, μ, reported by 45. For each panel, the posterior median slope (β) and 95% credible interval of the regression of $log(V_A)$ on $log(\pi)$ is indicated. Curves illustrate the expected relationship between $log(VA)$ and $log(\pi)$ for two extreme scenarios: variation in π is driven (i) only by changes in μ, with N_e fixed at 10^5 (black); and (ii) only by changes in N_e, with μ fixed at 0.7$\times 10^{-8}$ (red). Solid lines/circles indicate parameter combinations where the relevant approximation is expected to hold ($20L\mu \leq E[m^2]/V_S$ for HOC and $20L\mu \geq E[m^2]/V_S$ for the deterministic (i.e $N_e = \infty$) Gaussian) and dashed lines/open circles not. All predictions assume that the trait is determined by $n = 100$ unlinked loci of length $L = 1000$ with $V_S = 20$. The shaded region shows the range of pairwise nucleotide diversity for the species included in our analysis of nucleotide diversity vs. evolvability. The darker shaded region shows the range for species that are listed as Near Threatened, Threatened, Vulnerable, Endangered or Critically Endangered on the IUCN Red List.

such that $V_A(t) \propto \pi(t)$ in generations thereafter (assuming V_m and μ remain constant) and so the relationship between $ln(V_A(t))$ and $ln(\pi(t))$ will be one-to-one. While the condition $V_A(t-1) = \pi(t-1)V_m/2\mu$ seems restrictive, note that it is satisfied at equilibrium under neutrality since $\pi = 4N_e\mu$ and $V_A = 2V_mN_e$ such that $V_A = \pi V_m/2\mu$. Consequently, under neutrality a population only has to come to equilibrium once for the relationship between $ln(V_A(t))$ and $ln(\pi(t))$ to remain close to one-to-one. This relationship persists even if the population later departs from equilibrium due to changes in population size, and is only disrupted by external perturbations to allele frequencies (e.g. a translocation event). To illustrate this idea, in Figure S7 we iterate Equations 42 and 43 for a thousand generations with $N_e = 100$ and $\pi(0) = 0.01$. We do this for four parameter combinations with V_m equal to 0.001 or 0.0001 and μ equal to 10^{-8} or 10^{-6}. Since $\pi(0) = 0.01$ is greater than what would be expected given $N_e = 100$ and the mutation rates, $\pi(0)$ is not currently at equilibrium and so π is expected to decrease over time. For each parameter combination, two populations were iterated. One where V_A was initiated at its expected value given $\pi(0)$ ($V_A(0) = \pi(0)V_m/2\mu$: dashed lines) and one where V_A initiated at one (solid lines). In all populations, $V_A(0)$ is higher than what would be expected given values for V_m and $N_e = 100$. However, simulations where $V_A(0)$ is initiated at $\pi(0)V_m/2\mu$ are consistent with a scenario where V_A and π had come to their equilibrium at some previous point in time where N_e was higher. We refer to these populations as previously equilibrated. While we see non-linearity between $ln(V_A)$ and $ln(\pi)$ when sampled (over time) from populations that were never previously equilibrated, populations that were previously equilibrated show close to linear one-to-one relationships.

Non-equilibrium: Under stabilising selection. Selected loci are expected to equilibrate faster than neutral loci: in the extreme case lethals equilibrate in one generation. Consequently, when a trait is under selection, V_A may equilibrate at a faster rate than π. The recurrence equation under the Gaussian approximation is (50):

$$V_A(t) = V_A(t-1)\left[1 - \frac{1}{2N_e(t-1)} + \frac{1}{2n}\left(1 - \frac{1}{N_e(t-1)}\right)\frac{V_A(t-1)}{V_A(t-1)+V_S}\right] + V_m \quad [46]$$

and as V_S increases (i.e. the strength of stabilising selection diminishes) or n increases (i.e. the strength of stabilising selection per region diminishes) the recurrence equation tends to the neutral case. Note that the original derivation (50) is in terms of kh^2 where k is the proportional reduction in the phenotypic variance caused by stabilising selection and h^2 is the trait heritability: $kh^2 = V_A/(V_A + V_S)$. Setting $V_A(t-1) = \pi(t-1)V_m/2\mu$ as before, we have

$$\begin{aligned} V_A(t) = &\ \tfrac{V_m}{2\mu}\left(\pi(t-1)\left[1 - \tfrac{1}{2N_e(t-1)}\right] + 2\mu + \pi(t-1)\tfrac{1}{2n}\left(1 - \tfrac{1}{N_e(t-1)}\right)\tfrac{V_A(t-1)}{V_A(t-1)+V_S}\right) \\ V_A(t) = &\ \tfrac{V_m}{2\mu}\left(\pi(t) + \pi(t-1)\tfrac{1}{2n}\left(1 - \tfrac{1}{N_e(t-1)}\right)\tfrac{V_A(t-1)}{V_A(t-1)+V_S}\right) \end{aligned} \quad [47]$$

which shows that V_A will equilibrate faster than π when the strength of stabilising selection is strong and/or there are few loci contributing to V_A. In Figure S8 we initiate populations at their equilibrium values for π (Equation 23) and V_A (Equation 38) given an effective population size of 1,000 and then follow their trajectory following a bottleneck event where N_e instantaneously becomes 100. As in the neutral scenario above, we use four parameter combinations with V_m equal to 0.001 or 0.0001 and μ equal to 10^{-8} or 10^{-6}. For each parameter combination we iterate Equations 47 and 43 when the number of loci is either 100 (selection per locus is weak: solid black lines) or 10 (selection per locus is strong: solid red lines). When selection per locus is weak the relationship is close to linear with a slope of one reflecting the neutral case, but when selection per locus is strong the relationship between $ln(V_A)$ and $ln(\pi)$ becomes weaker, particularly if V_m is large.

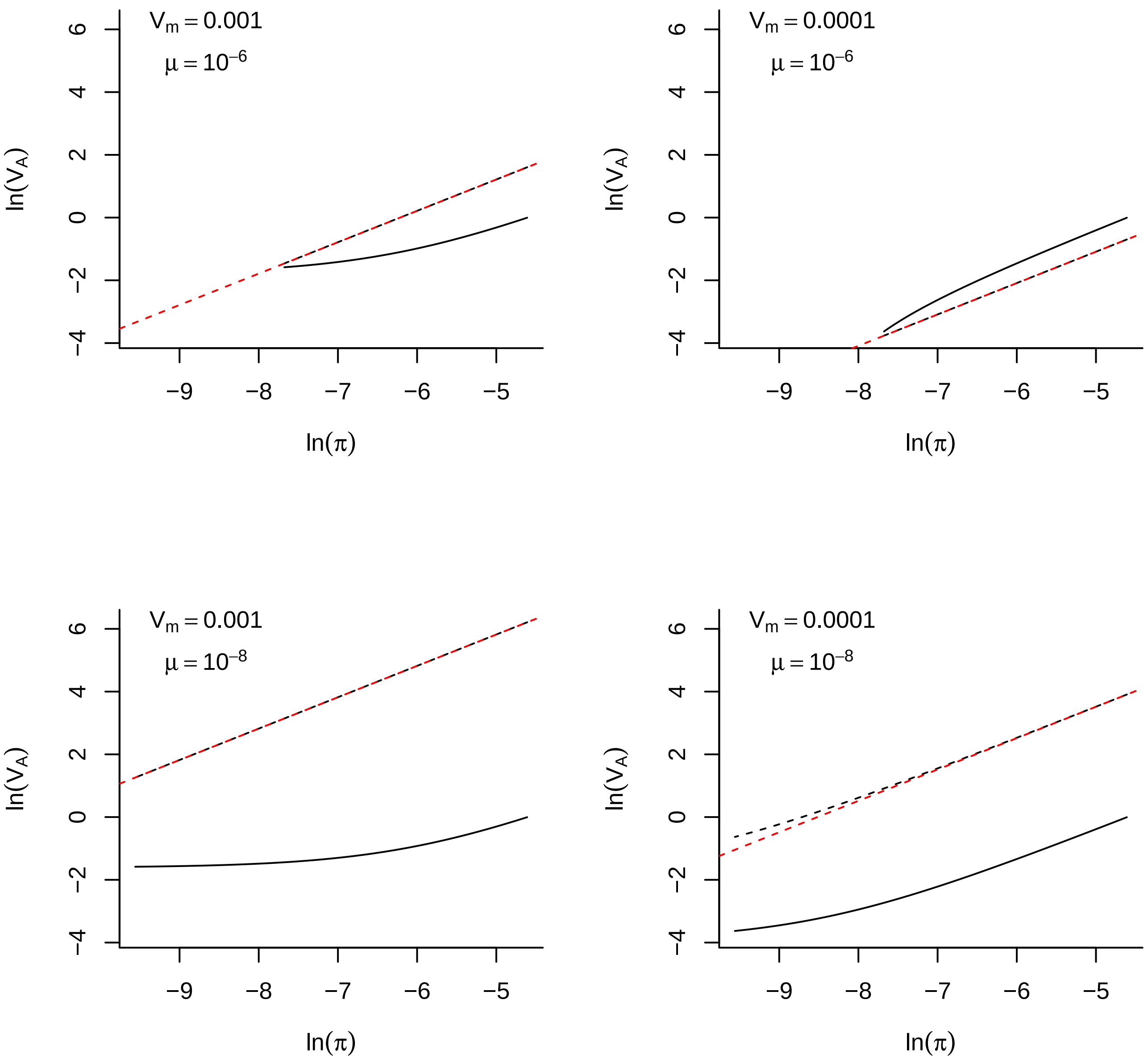

Fig. S7. Populations are initialised with a genetic diversity, $\pi(0)$, of 0.01. Solid black lines are for populations where the additive genetic variance, $V_A(0)$, was initialised at one (not previously equilibrated) and the dashed black lines are for previously equilibrated populations where $V_A(0) = \pi(0)V_m/2\mu$. Note that in all cases all quantities are far above their expected equilibrium values given $N_e = 100$ and so the quantities are moving from right to left over time, representing the loss of genetic variation following a bottleneck. Nevertheless, in previously equilibrated populations, $ln(V_A)$ has a close to linear dependence on $ln(\pi)$ with a slope of one and intercept of $ln(V_m/2\mu)$ as would be seen in permanently equilibrated populations (red dashed lines).

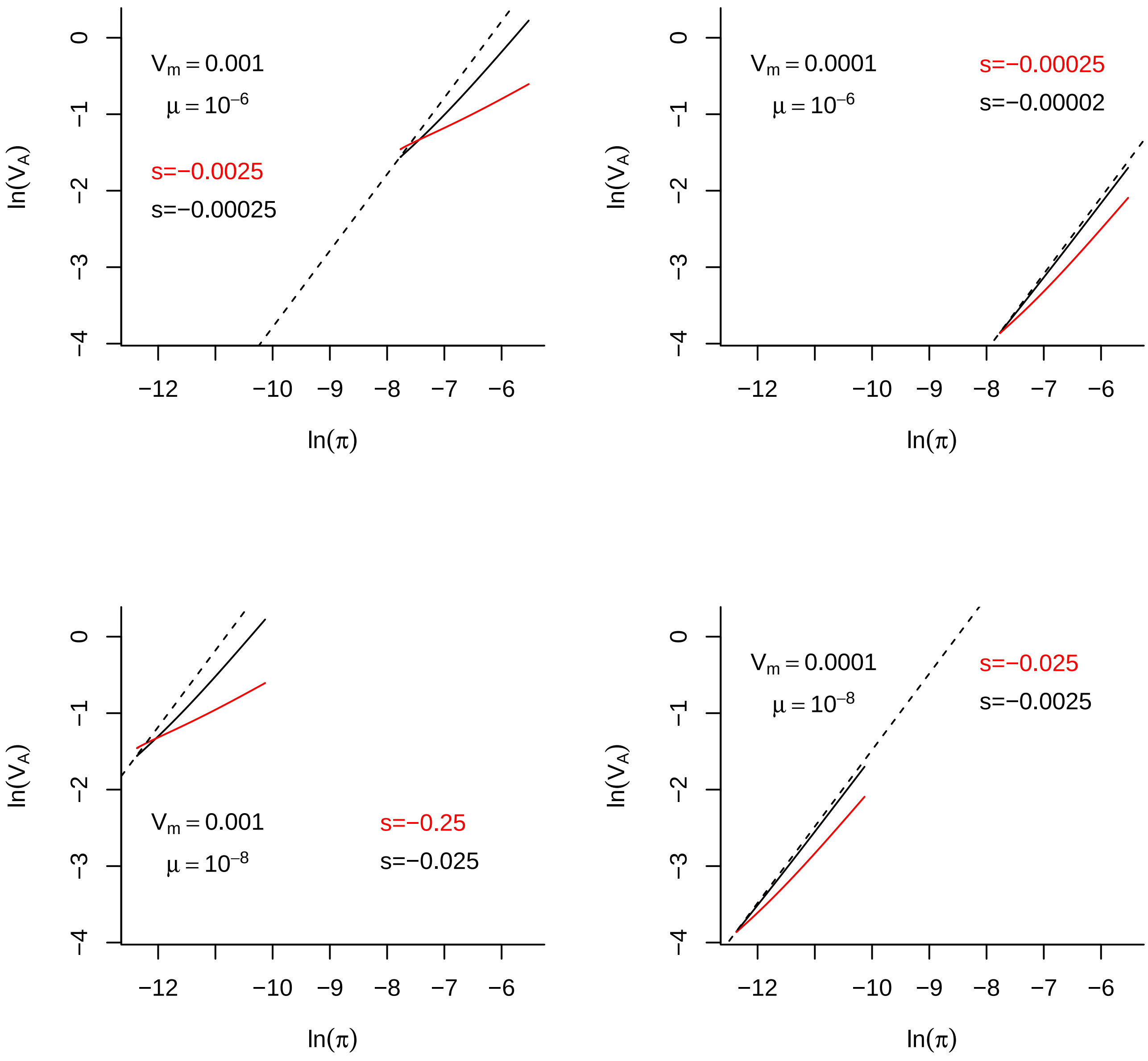

Fig. S8. Populations are initialised with a genetic diversity ($\pi(0)$) equal to the neutral expectation when $N_e = 1,000$. Similarly, the additive genetic variance is initialised ($V_A(0)$) at its Gaussian mutation-selection-drift balance expectation. Following initialisation, populations experience a bottleneck event where N_e instantaneously becomes 100. We follow their trajectory when the number of loci contributing to the trait is large ($n = 100$) and so selection per-locus is weak (solid black line) and when the number of loci is small ($n = 10$) and so selection per-locus is strong (solid red line). Different scenarios involve different combinations of V_m and μ but V_s=20 and L=1,000 are constant. Note that in the neutral case these initial conditions would give rise to a relationship that is close to one-to-one with an intercept of $ln(V_m/2\mu)$ (dashed black line - See Figure S7).

Table S5. Summary of fixed effects (posterior mean, lower and upper 95% credible intervals) from the univariate model of evolvability estimates, $ln(I_A)$. pMCMC is twice the posterior probability that the effect is less than or greater than zero (whichever is smaller) and $P(>\chi^2)$ is the P-value of an omnibus test (a Wald test using the posterior mean and covariance matrix) for multi-category factors (displayed next to the final level). The intercept corresponds to linear morphological traits measured using a pedigree-based (animal model) estimate of relatedness.

Parameter	mean	l-95%	u-95%	pMCMC	$P(>\chi^2)$
Intercept	-0.149	-2.628	2.302	0.877	
$\ln(\hat{\pi})$	0.162	-0.125	0.452	0.282	
Trait type: fitness	-0.665	-2.289	0.865	0.403	
Trait type: life history	0.124	-0.570	0.873	0.690	
Trait type: behaviour	1.488	0.561	2.437	0.004	
Trait type: physiological	0.312	-0.200	0.822	0.239	0.018
Method: mid-parent-offspring	-1.041	-2.148	-0.073	0.052	
Method: full-sib	0.100	-0.785	1.047	0.841	
Method: half-sib	0.250	-0.542	0.986	0.511	
Method: clonal	0.317	-0.654	1.252	0.518	
Method: realized	-0.423	-2.143	1.307	0.624	
Method: single-parent-offspring	0.062	-0.780	0.972	0.891	0.383
number of fixed predictors	0.011	-0.103	0.127	0.852	
number of random predictors	0.326	0.006	0.640	0.042	
Dimension: quadratic	0.740	-0.030	1.551	0.068	
Dimension: cubic	0.996	0.518	1.566	$<0.5\times10^{-3}$	
Dimension: meristic	1.360	0.740	2.045	10^{-3}	
Dimension: time	0.335	-0.487	1.116	0.397	
Dimension: other	0.456	-0.109	0.984	0.122	< 0.001

Table S6. Summary of estimated variance components (posterior mode, lower and upper 95% credible intervals) from the univariate model of evolvability estimates, $ln(I_A)$, across 108 species in which $ln(\hat{\pi})$ was fitted as a predictor with regression slope, β. Variance in evolvability attributable to variance in $ln(\hat{\pi})$ was calculated as $\beta^2 VAR(ln(\hat{\pi}))$. True between-species variance is the sum of the *phylogenetic*, *non-phylogenetic species* and $ln(\hat{\pi})$ variance components. Total variance is the sum of all reported variance components after conditioning on fixed effects.

	Variance			% of between-species variance			% of total variance		
**Variance component**	mode	l-95%	u-95%	mode	l-95%	u-95%	mode	l-95%	u-95%
ln($\hat{\pi}$)	0.407×10^{-3}	0.21×10^{-6}	0.194	0.048	0.127×10^{-4}	8.153	0.009	0.397×10^{-5}	2.580
Phylogenetic species	1.580	0.182	5.944	98.304	51.558	99.987	30.668	6.441	58.792
Non-phylogenetic species	0.002	0.397×10^{-8}	0.630	0.169	0.964×10^{-7}	45.401	0.055	0.429×10^{-7}	10.194
Trait	1.011	0.760	1.486				16.999	8.870	23.901
Publication	1.291	0.885	2.003				17.200	9.766	31.077
Residual	1.855	1.677	2.068				25.570	15.909	37.679
Between-species	1.806	0.603	6.200				30.680	14.228	61.255
Total	6.184	4.743	10.505						

Table S7. Summary of fixed effects (posterior mean, lower and upper 95% credible intervals) from the univariate model of heritability estimates, h^2. pMCMC is twice the posterior probability that the effect is less than or greater than zero (whichever is smaller) and $P(>\chi^2)$ is the P-value of an omnibus test (a Wald test using the posterior mean and covariance matrix) for multi-category factors (displayed next to the final level). The intercept corresponds to linear morphological traits measured using a pedigree-based (animal model) estimate of relatedness.

Parameter	mean	l-95%	u-95%	pMCMC	$P(>\chi^2)$
Intercept	0.265	0.032	0.525	0.024	
$\ln(\hat{\pi})$	-0.022	-0.055	0.011	0.193	
Trait type: fitness	-0.149	-0.331	0.022	0.091	
Trait type: life history	-0.041	-0.093	0.016	0.148	
Trait type: behaviour	-0.104	-0.185	-0.031	0.010	
Trait type: physiological	-0.055	-0.102	-0.013	0.016	0.018
Method: mid-parent-offspring	-0.012	-0.110	0.086	0.813	
Method: full-sib	0.096	-0.004	0.188	0.050	
Method: half-sib	0.019	-0.070	0.103	0.647	
Method: clonal	0.079	-0.014	0.179	0.123	
Method: realized	0.144	-0.016	0.321	0.090	
Method: single-parent-offspring	0.072	-0.003	0.153	0.078	0.185
number of fixed predictors	-0.008	-0.021	0.003	0.223	
number of random predictors	-0.034	-0.063	-0.007	0.022	
Dimension: quadratic	0.032	-0.059	0.110	0.448	
Dimension: cubic	0.010	-0.044	0.064	0.745	
Dimension: meristic	0.007	-0.055	0.064	0.831	
Dimension: time	0.009	-0.063	0.078	0.767	
Dimension: other	-0.028	-0.069	0.010	0.178	0.432

Table S8. Summary of estimated variance components (posterior mode, lower and upper 95% credible intervals) from the univariate model of heritability estimates, h^2, across 130 species in which $ln(\hat{\pi})$ was fitted as a predictor with regression slope, β. Variance in heritability attributable to variance in $ln(\hat{\pi})$ was calculated as $\beta^2 VAR(ln(\hat{\pi}))$. True between-species variance is the sum of the *phylogenetic*, *non-phylogenetic species* and $ln(\hat{\pi})$ variance components. Total variance is the sum of all reported variance components after conditioning on fixed effects.

	Variance			% of between-species variance			% of total variance		
**Variance component**	mode	l-95%	u-95%	mode	l-95%	u-95%	mode	l-95%	u-95%
ln($\hat{\pi}$)	0.159$\times 10^{-4}$	0.259$\times 10^{-8}$	0.003	0.063	0.55$\times 10^{-5}$	21.254	0.019	0.224$\times 10^{-5}$	3.589
Phylogenetic species	0.507$\times 10^{-3}$	0.336$\times 10^{-8}$	0.076	0.429	0.413$\times 10^{-4}$	97.692	0.328	0.408$\times 10^{-5}$	50.756
Non-phylogenetic species	0.009	0.198$\times 10^{-6}$	0.015	0.600	0.366$\times 10^{-3}$	92.463	8.585	0.176$\times 10^{-3}$	16.790
Trait	0.009	0.007	0.013				9.423	4.710	14.001
Publication	0.018	0.014	0.028				23.397	11.849	31.408
Residual	0.040	0.038	0.043				46.687	25.672	54.280
Between-species	0.011	0.005	0.078				17.373	7.079	53.078
Total	0.081	0.073	0.152						

Table S9. Number of species with estimates of evolvability, I_A, or heritability, h^2, grouped by IUCN Red List status

	Number of Species	
**IUCN status**	**Evolvability**	**Heritability**
Least concern	100	124
Near threatened	5	5
Vulnerable	7	10
Endangered	3	3
Critically Endangered	2	4
Not classified	76	100

Table S10. Summary of fixed effects (posterior mean, lower and upper 95% credible intervals) from the univariate model of evolvability estimates, $ln(I_A)$, with IUCN Red List status included as a predictor. pMCMC is twice the posterior probability that the effect is less than or greater than zero (whichever is smaller) and $P(>\chi^2)$ is the P-value of an omnibus test (a Wald test using the posterior mean and covariance matrix) for multi-category factors (displayed next to the final level). The intercept corresponds to linear morphological traits measured using a pedigree-based (animal model) estimate of relatedness.

Parameter	mean	l-95%	u-95%	pMCMC	$P(>\chi^2)$
Intercept	-1.226	-3.597	1.274	0.245	
IUCN: near threatened	0.223	-0.993	1.264	0.668	
IUCN: vulnerable	-0.103	-1.134	1.066	0.865	
IUCN: endangered	-0.023	-1.305	1.206	0.975	
IUCN: critically endangered	0.310	-1.039	1.702	0.668	0.992
Trait type: fitness	0.207	-1.363	1.911	0.827	
Trait type: life history	0.198	-0.523	0.996	0.648	
Trait type: behaviour	1.694	0.931	2.532	$<0.5\times10^{-3}$	
Trait type: physiological	0.250	-0.304	0.854	0.372	0.002
Method: mid-parent-offspring	-1.254	-2.220	-0.246	0.023	
Method: full-sib	0.826	-0.255	1.903	0.148	
Method: half-sib	1.031	-0.008	2.070	0.055	
Method: clonal	1.093	-0.121	2.459	0.103	
Method: realized	2.448	-0.178	4.937	0.062	
Method: single-parent-offspring	0.389	-0.465	1.291	0.400	0.004
number of fixed predictors	0.027	-0.073	0.136	0.620	
number of random predictors	0.247	-0.013	0.490	0.047	
Dimension: quadratic	0.906	0.052	1.762	0.040	
Dimension: cubic	1.046	0.493	1.559	$<0.5\times10^{-3}$	
Dimension: meristic	1.247	0.573	1.877	$<0.5\times10^{-3}$	
Dimension: time	-0.086	-0.973	0.698	0.839	
Dimension: other	0.505	-0.053	1.102	0.088	<0.001

Table S11. Summary of fixed effects (posterior mean, lower and upper 95% credible intervals) from the univariate model of heritability estimates, h^2, with IUCN Red List status included as a predictor. pMCMC is twice the posterior probability that the effect is less than or greater than zero (whichever is smaller) and $P(>\chi^2)$ is the P-value of an omnibus test (a Wald test using the posterior mean and covariance matrix) for multi-category factors (displayed next to the final level). The intercept corresponds to linear morphological traits measured using a pedigree-based (animal model) estimate of relatedness.

Parameter	mean	l-95%	u-95%	pMCMC	$P(>\chi^2)$
Intercept	0.319	-0.056	0.603	0.077	
IUCN: near threatened	-0.002	-0.133	0.142	0.990	
IUCN: vulnerable	0.157	0.017	0.290	0.020	
IUCN: endangered	0.070	-0.101	0.237	0.409	
IUCN: critically endangered	0.065	-0.097	0.230	0.447	0.179
Trait type: fitness	-0.137	-0.288	0.045	0.119	
Trait type: life history	-0.022	-0.087	0.030	0.475	
Trait type: behaviour	-0.076	-0.149	-0.006	0.041	
Trait type: physiological	-0.107	-0.152	-0.063	$<0.5\times10^{-3}$	<0.001
Method: mid-parent-offspring	0.003	-0.089	0.091	0.956	
Method: full-sib	0.223	0.117	0.332	$<0.5\times10^{-3}$	
Method: half-sib	-0.001	-0.122	0.122	0.991	
Method: clonal	0.245	0.122	0.387	$<0.5\times10^{-3}$	
Method: realized	0.068	-0.169	0.278	0.552	
Method: single-parent-offspring	0.095	0.022	0.167	0.005	0.001
number of fixed predictors	-0.009	-0.020	0.002	0.096	
number of random predictors	-0.029	-0.052	-0.006	0.010	
Dimension: quadratic	0.006	-0.081	0.114	0.921	
Dimension: cubic	0.002	-0.049	0.053	0.939	
Dimension: meristic	-0.034	-0.091	0.025	0.242	
Dimension: time	-0.005	-0.073	0.064	0.872	
Dimension: other	-0.026	-0.067	0.009	0.178	0.671

Table S12. Summary of estimated fixed effects (posterior mean, lower and upper 95% credible intervals) from the bivariate model of evolvability, $ln(I_A)$, and nucleotide diversity estimates, $ln(\hat{\pi})$. Intercepts are estimated for both responses, whereas additional fixed effects are specified only for the evolvability response. pMCMC is twice the posterior probability that the effect is less than or greater than zero (whichever is smaller) and $P(>\chi^2)$ is the P-value of an omnibus test (a Wald test using the posterior mean and covariance matrix) for multi-category factors (displayed next to the final level). The intercept for $log(I_A)$ corresponds to linear morphological traits measured using a pedigree-based (animal model) estimate of relatedness.

Parameter	mean	l-95%	u-95%	pMCMC	$P(>\chi^2)$
Intercept: $\ln(\hat{\pi})$	-0.984	-3.044	0.795	0.301	
Intercept: $\ln(I_A)$	-5.171	-6.642	-3.960	$<0.5\times10^{-3}$	
Trait type: fitness	-0.241	-1.739	1.203	0.741	
Trait type: life history	0.170	-0.406	0.747	0.562	
Trait type: behaviour	1.707	0.973	2.427	$<0.5\times10^{-3}$	
Trait type: physiological	0.340	-0.118	0.842	0.173	< 0.001
Method: mid-parent-offspring	-1.077	-1.880	-0.230	0.011	
Method: full-sib	0.525	-0.210	1.335	0.184	
Method: half-sib	0.223	-0.361	0.823	0.432	
Method: clonal	0.384	-0.479	1.170	0.372	
Method: realized	-0.563	-1.913	0.790	0.447	
Method: single-parent-offspring	0.088	-0.618	0.743	0.779	0.070
number of fixed predictors	0.010	-0.078	0.098	0.809	
number of random predictors	0.159	-0.065	0.385	0.162	
Dimension: quadratic	0.497	-0.167	1.126	0.137	
Dimension: cubic	1.120	0.651	1.550	$<0.5\times10^{-3}$	
Dimension: meristic	1.349	0.857	1.890	$<0.5\times10^{-3}$	
Dimension: time	0.361	-0.260	1.048	0.276	
Dimension: other	0.498	-0.008	0.981	0.045	< 0.001

Table S13. Summary of estimated variance components (posterior mode, lower and upper 95% credible intervals) from the bivariate model of evolvability, $ln(I_A)$, and nucleotide diversity, $ln(\hat{\pi})$, estimates across 193 species. The variance in $ln(I_A)$ attributable to $ln(\hat{\pi})$ was calculated from their (co)variance.

		Variance			% of between-species variance			% of total variance		
**At level**	**Variance component**	mode	l-95%	u-95%	mode	l-95%	u-95%	mode	l-95%	u-95%
$ln(\widehat{I_A})$	ln($\hat{\pi}$)	0.004	0.522×10^{-8}	0.815	0.093	0.157×10^{-6}	18.799	0.056	0.703×10^{-7}	9.140
	Phylogenetic species	2.470	0.477	6.022	89.720	62.063	97.224	36.427	12.790	58.177
	Non-phylogenetic species	0.197	0.086	0.573	5.265	1.104	32.474	2.883	1.012	8.952
	Trait	0.954	0.705	1.251				12.625	7.675	19.780
	Publication	1.078	0.704	1.513				13.106	7.484	23.690
	Residual	2.072	1.919	2.256				30.500	17.764	39.946
	Between-species	2.783	0.819	6.583				41.580	20.330	63.886
	Total	6.338	5.033	10.820						
$ln(\hat{\pi})$	Phylogenetic species	0.678	0.174	2.905						
	Non-phylogenetic species	0.766	0.556	1.066						

Table S14. Summary of estimated fixed effects (posterior mean, lower and upper 95% credible intervals) from the bivariate model of heritability, h^2, and nucleotide diversity estimates, $ln(\hat{\pi})$. Intercepts are estimated for both responses, whereas additional fixed effects are specified only for the heritability response. pMCMC is twice the posterior probability that the effect is less than or greater than zero (whichever is smaller) and $P(>\chi^2)$ is the P-value of an omnibus test (a Wald test using the posterior mean and covariance matrix) for multi-category factors (displayed next to the final level). The intercept for h^2 corresponds to linear morphological traits measured using a pedigree-based (animal model) estimate of relatedness.

Parameter	mean	l-95%	u-95%	pMCMC	$P(>\chi^2)$
Intercept: $\ln(\hat{\pi})$	0.376	0.295	0.440	$<0.5\times10^{-3}$	
Intercept: h^2)	-4.888	-6.210	-3.688	$<0.5\times10^{-3}$	
Trait type: fitness	-0.164	-0.303	-0.008	0.035	
Trait type: life history	-0.033	-0.073	0.010	0.131	
Trait type: behaviour	-0.092	-0.154	-0.035	0.002	
Trait type: physiological	-0.080	-0.119	-0.042	$<0.5\times10^{-3}$	<0.001
Method: mid-parent-offspring	0.035	-0.049	0.112	0.405	
Method: full-sib	0.117	0.030	0.197	0.004	
Method: half-sib	0.147×10^{-3}	-0.063	0.069	0.984	
Method: clonal	0.083	-0.005	0.172	0.069	
Method: realized	0.147	0.003	0.272	0.035	
Method: single-parent-offspring	0.074	0.008	0.132	0.015	0.016
number of fixed predictors	-0.013	-0.023	-0.004	0.010	
number of random predictors	-0.025	-0.048	-0.004	0.024	
Dimension: quadratic	0.003	-0.063	0.080	0.930	
Dimension: cubic	-0.011	-0.053	0.028	0.578	
Dimension: meristic	-0.023	-0.068	0.022	0.347	
Dimension: time	-0.494×10^{-4}	-0.054	0.052	0.990	
Dimension: other	-0.039	-0.073	-0.008	0.021	0.166

Table S15. Summary of estimated variance components (posterior mode, lower and upper 95% credible intervals) from the bivariate model of heritability, h^2, and nucleotide diversity, $ln(\hat{\pi})$, estimates across 246 species. The variance in h^2 attributable to $ln(\hat{\pi})$ was calculated from their (co)variance.

		Variance			% of between-species variance			% of total variance		
**At level**	**Variance component**	mode	l-95%	u-95%	mode	l-95%	u-95%	mode	l-95%	u-95%
h^2	ln($\hat{\pi}$)	0.252×10^{-4}	0.818×10^{-10}	0.002	0.044	0.376×10^{-6}	7.474	0.013	0.932×10^{-7}	2.394
	Phylogenetic species	0.85×10^{-4}	0.111×10^{-8}	0.008	0.126	0.618×10^{-5}	24.605	0.021	0.139×10^{-5}	8.375
	Non-phylogenetic species	0.025	0.018	0.032	99.627	70.926	99.999	26.614	20.490	32.682
	Trait	0.006	0.004	0.008				6.893	4.436	8.999
	Publication	0.018	0.014	0.024				19.927	14.738	24.882
	Residual	0.040	0.038	0.043				44.854	39.159	49.482
	Between-species	0.026	0.018	0.037				29.831	22.222	36.895
	Total	0.091	0.082	0.102						
$ln(\hat{\pi})$	Phylogenetic species	1.023	0.226	2.791						
	Non-phylogenetic species	0.846	0.568	1.063						

References

1. D Houle, C Pélabon, GP Wagner, TF Hansen, Measurement and meaning in biology. *The quarterly review biology* **86**, 3–34 (2011).
2. TF Hansen, C Pélabon, D Houle, Heritability is not evolvability. *Evol. Biol.* **38**, 258–277 (2011).
3. C Pélabon, CH Hilde, S Einum, M Gamelon, On the use of the coefficient of variation to quantify and compare trait variation. *Evol. Lett.* **4**, 180–188 (2020).
4. D Houle, Comparing evolvability and variability of quantitative traits. *Genetics* **130**, 195–204 (1992).
5. EA Mittell, S Nakagawa, JD Hadfield, Are molecular markers useful predictors of adaptive potential? *Ecol. letters* **18**, 772–778 (2015).
6. EA Young, E Postma, Low interspecific variation and no phylogenetic signal in additive genetic variance in wild bird and mammal populations. *Ecol. Evol.* **13**, e10693 (2023).
7. DS Falconer, TF Mackay, *Introduction To Quantitative Genetics.* (Longman, Harlow, UK), 4th edition, (1996).
8. AJ Wilson, Why h2 does not always equal VA/VP? *J. Evol. Biol.* **21**, 647–650 (2008).
9. SW Raudenbush, AS Bryk, Examining correlates of diversity. *J. Educ. Stat.* **12**, 241–269 (1987).
10. S Nakagawa, et al., Meta-analysis of variation: ecological and evolutionary applications and beyond. *Methods Ecol. Evol.* **6**, 143–152 (2015).
11. S Searle, Matrix methods in components of variance and covariance analysis. *The Annals Math. Stat.* pp. 737–748 (1956).
12. JG King, JL Pick, JD Hadfield, Quantifying the correlation between variance components: An extension to the double-hierarchical generalised linear model. *Methods Ecol. Evol.* **16**, 2345–2361 (2025).
13. The Darwin Tree of Life Project Consortium, Sequence locally, think globally: The Darwin Tree of Life Project. *Proc. Natl. Acad. Sci.* **119**, e2115642118 (2022).
14. TR Ranallo-Benavidez, KS Jaron, MC Schatz, GenomeScope 2.0 and Smudgeplot for reference-free profiling of polyploid genomes. *Nat. Commun.* **11**, 1432 (2020).
15. MD Roberts, EB Josephs, k-mer-based diversity scales with population size proxies more than nucleotide diversity in a meta-analysis of 98 plant species. *Evol. Lett.* **9**, 434–445 (2025).
16. H Schielzeth, et al., Robustness of linear mixed-effects models to violations of distributional assumptions. *Methods ecology evolution* **11**, 1141–1152 (2020).
17. JL Pick, HE Lemon, CE Thomson, JD Hadfield, Decomposing phenotypic skew and its effects on the predicted response to strong selection. *Nat. Ecol. & Evol.* **6**, 774–785 (2022).
18. J Berkson, Are there two regressions? *J. american statistical association* **45**, 164–180 (1950).
19. JD Hadfield, MCMC Methods for Multi-Response Generalized Linear Mixed Models: The MCMCglmm R Package. *J. statistical software* **33**, 1–22 (2010).
20. R Core Team, *R: A Language and Environment for Statistical Computing* (R Foundation for Statistical Computing, Vienna, Austria), (2023).
21. RH Baayen, DJ Davidson, DM Bates, Mixed-effects modeling with crossed random effects for subjects and items. *J. memory language* **59**, 390–412 (2008).
22. JL Pick, et al., Describing posterior distributions of variance components: Problems and the use of null distributions to aid interpretation. *Methods Ecol. Evol.* **14**, 2557–2574 (2023).
23. DJ Benjamin, et al., Redefine statistical significance. *Nat. Hum. Behav.* **2**, 6–10 (2018).
24. Stan Development Team, Rstan: the R interface to Stan (2025) R package version 2.32.7.
25. S Kumar, et al., TimeTree 5: An expanded resource for species divergence times. *Mol. biology evolution* **39**, msac174 (2022).
26. FW Allendorf, et al., *Conservation and the Genomics of Populations.* (Oxford University Press, Oxford, New York), Third edition, (2022).
27. VL Corre, Variation at two flowering time genes within and among populations of Arabidopsis thaliana: Comparison with markers and traits. *Mol. Ecol.* **14**, 4181–4192 (2005).
28. C Schmidt, S Hoban, M Hunter, I Paz-Vinas, CJ Garroway, Genetic diversity and IUCN Red List status. *Conserv. Biol.* **37**, e14064 (2023).
29. CM McLaughlin, C Hinshaw, S Sandoval-Arango, M Zavala-Paez, JA Hamilton, Redlisting genetics: Towards inclusion of genetic data in IUCN Red List assessments. *Conserv. Genet.* **26**, 213–223 (2025).
30. JC Teixeira, CD Huber, The inflated significance of neutral genetic diversity in conservation genetics. *Proc. Natl. Acad. Sci.* **118**, e2015096118 (2021).
31. IUCN, The IUCN Red List of Threatened Species. Version 2025-1 (https://www.iucnredlist.org) (2025) [Accessed 13 August 2025].
32. CE Thomson, et al., Selection on parental performance opposes selection for larger body mass in a wild population of blue tits. *Evolution* **71**, 716–732 (2017).
33. B Walsh, M Lynch, eds., *Evolution and Selection of Quantitative Traits.* (Oxford University Press), (2018).
34. G Clayton, A Robertson, Mutation and quantitative variation. *The Am. Nat.* **89**, 151–158 (1955).
35. M Turelli, Heritable genetic variation via mutation-selection balance: Lerch's zeta meets the abdominal bristle. *Theor. population biology* **25**, 138–193 (1984).
36. BDH Latter, Natural selection for an intermediate optimum. *Aust. J. Biol. Sci.* **13**, 30–35 (1960).

37. BDH Latter, Selection in finite populations with multiple alleles. II. Centripetal selection, mutation, and isoallelic variation. *Genetics* **66**, 165–186 (1970).
38. M Kimura, A stochastic model concerning the maintenance of genetic variability in quantitative characters. *Proc. Natl. Acad. Sci.* **54**, 731–736 (1965).
39. R Lande, The response to selection on major and minor mutations affecting a metrical trait. *Heredity* **50**, 47–65 (1983).
40. M Lynch, et al., The divergence of mutation rates and spectra across the Tree of Life. *The EMBO Reports* **24**, EMBR202357561 (2023).
41. R Bürger, GP Wagner, F Stettinger, How much heritable variation can be maintained in finite populations by mutation–selection balance? *Evolution* **43**, 1748–1766 (1989).
42. T Johnson, N Barton, Theoretical models of selection and mutation on quantitative traits. *Philos. Transactions Royal Soc. B: Biol. Sci.* **360**, 1411–1425 (2005).
43. D Houle, The maintenance of polygenic variation in finite populations. *Evolution* **43**, 1767–1780 (1989).
44. YB Simons, et al., Simple scaling laws control the genetic architectures of human complex traits. *PLOS Biol.* **23**, e3003402 (2025).
45. L Lewin, A Eyre-Walker, A comparative analysis of long-term effective population sizes across eukaryotes. *Mol. Ecol.* **35**, e70265 (2026).
46. R Lande, GF Barrowclough, Effective population size, genetic variation, and their use in population management in *Viable Populations for Conservation*, ed. ME Soulé. (Cambridge University Press, Cambridge), pp. 87–124 (1987).
47. M Lynch, A quantitative-genetic perspective on conservation issues in *Conservation Genetics: Case Histories From Nature*, eds. JC Avise, JL Hamrick. (Chapman and Hall, New York), pp. 471–501 (1996).
48. M Lynch, WG Hill, Phenotypic evolution by neutral mutation. *Evolution* **40**, 915–935 (1986).
49. G Malécot, *The Mathematics of Heredity.* (Masson & Cie, Paris), (1948).
50. C Chevalet, An approximate theory of selection assuming a finite number of quantitative trait loci. *Genet. Sel. Evol.* **26**, 379 (1994).
